# Association of sugar intake from different sources with incident dementia in the prospective cohort of UK Biobank participants

**DOI:** 10.1186/s12937-023-00871-8

**Published:** 2023-09-04

**Authors:** Sylva M. Schaefer, Anna Kaiser, Gerrit Eichner, Mathias Fasshauer

**Affiliations:** 1 Institute of Nutritional Science, Justus-Liebig University of Giessen, 35390 Giessen, Germany; 2 Mathematical Institute, Justus-Liebig University of Giessen, Giessen, Germany; 3 Center for Sustainable Food Systems, Justus-Liebig University of Giessen, Giessen, Germany

**Keywords:** Carbohydrates, Dementia, Sugar, UK Biobank

## Abstract

**Background:**

Dementia is a common disease with around 55 million cases worldwide. Therefore, dietary changes and lifestyle interventions are important approaches to delay the progress of a decline in cognitive function. The study aims to explore the association of various sources of free sugars (FS) and intrinsic sugars with dementia risk in the prospective population-based UK Biobank cohort.

**Methods:**

Sugar consumption was assessed in 186,622 UK Biobank participants with at least one web-based dietary questionnaire (Oxford WebQ). Over a mean follow-up of 10.6 (standard deviation 1.1) years, 1498 incident dementia cases occurred. The hazard ratios (HR) for incident dementia were assessed with Cox proportional hazard regression models including sugar intake from different sources as penalized cubic splines to allow for non-linear predictor effects.

**Results:**

The intake of FS and intrinsic sugar was significantly associated with dementia risk in a J-shaped fashion with the HR-nadir observed at 9% and 8% total energy (%E), respectively. FS in beverages were significantly associated with dementia risk in an ascending approximately linear way, whereas no significant association was found for FS in solids. Assessing beverage subtypes, FS in soda/fruit drinks, milk-based drinks and to a lesser extent in juice were significantly and positively related to dementia risk, whereas no association was found for FS in tea/coffee. The association between sugar subtype consumption and dementia risk remained consistent in most sensitivity analyses but changed from a J-shape to a more linear shape when the analysis was restricted to participants with at least two Oxford WebQs.

**Conclusions:**

A linear-shaped association between sugar subtype intake and dementia risk is most consistently found for FS in beverages and more specifically for FS in soda/fruit drinks, as well as in milk-based drinks.

**Graphical Abstract:**

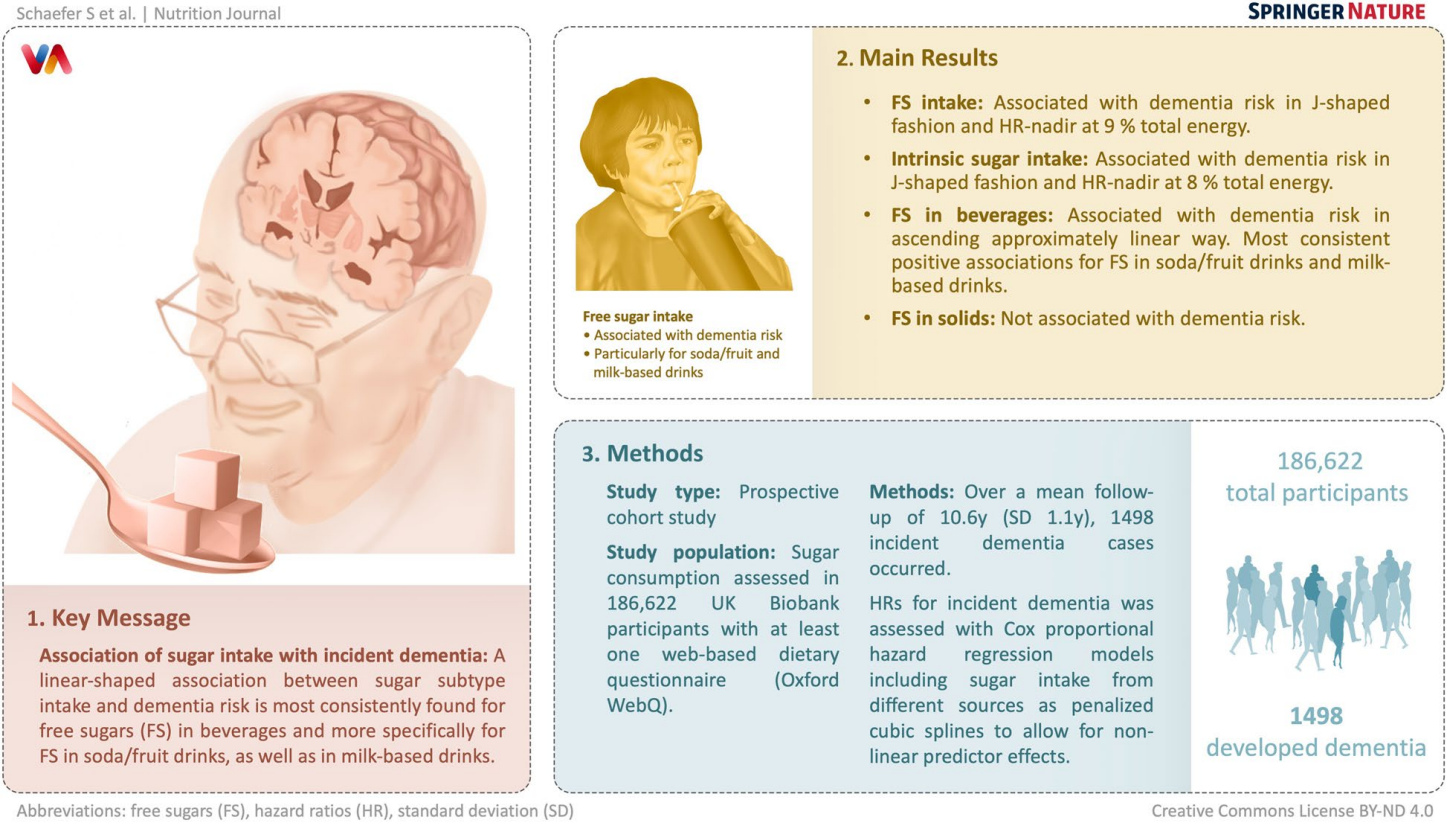

**Supplementary Information:**

The online version contains supplementary material available at 10.1186/s12937-023-00871-8.

## Background

Dementia is a common disease with around 55 million cases worldwide and almost 10 million new cases every year [1]. It is characterized by a deterioration in cognitive function beyond the usual effects of biological aging [1]. Even though age is one of the most important risks factors for dementia [1], there is evidence that being overweight [2] and obese [3] in midlife is similarly associated with a higher disease risk. Despite a large amount of research that has been conducted over the past decades, no effective pharmacological treatment for the main dementia types has been developed to date [4]. Therefore, dietary changes and lifestyle interventions remain important approaches, not only to delay the progress of a decline in cognitive function [5], but also to combat overweight and obesity [6].

A common approach to decrease body weight, and improve glucose control, as well as low-grade inflammation, is to follow a diet low in carbohydrates [7, 8]. Interestingly, studies on these diets provide promising results for the treatment and prevention of dementia [9]. However, long-term adherence to low carbohydrate diets is difficult to maintain because of severe limitations in the diversity of food choices [10]. In addition, various food items that are associated with better cognitive performance such as fruits, vegetables, legumes, and whole grains might also be excluded from the diet [11, 12]. Therefore, recent interventions have investigated the reduction of specific carbohydrate subtypes with a specific focus on limiting sugars [13, 14].

Sugars are defined as all mono- and disaccharides [15] and according to the World Health Organization (WHO), they can be divided into free sugars (FS) and intrinsic sugars [16]. FS are added to foods by the manufacturer, cook, or consumer, in addition to sugars naturally present in honey, syrups, and fruit juices [16]. The WHO recommends limiting FS to less than 10% of total energy intake and ideally to less than 5%, i.e., 50 g and 25 g FS per day, respectively, for a 2000 kcal diet [16]. In agreement with WHO recommendations, the National Health Service England (NHS) limits FS intake to less than 30 g per day for adults [17].

No study so far has systematically evaluated the association between FS consumption from various sources, including FS in beverages, beverage subtypes, solid foods, and solids subtypes on the one hand, and dementia risk on the other hand. To address this open point, all major FS sources summarized in Additional file 1 Fig. S1 were analysed concerning incident dementia in a large, well-characterized population of 186,622 UK Biobank participants.

We hypothesized that the link between FS and incident dementia depends on the FS source with a positive association found for beverages but not for solid foods similar to recent findings from our group studying all-cause mortality [18] and incident depression [19]. Furthermore, the link between intrinsic sugars, i.e., all sugars that are not FS including sugars from fruit, vegetables, and lactose in dairy products [16], and dementia risk is assessed for the first time.

## Methods

### Study design and participants

The UK Biobank study is a prospective cohort study that recruited more than half a million participants aged 37 to 73 across the UK between 2006 and 2010 [20]. Participants who filled out at least one web-based dietary questionnaire for the assessment of the previous 24 h dietary intakes (Oxford WebQ) [21] were selected for the current report (Additional file 1 Fig. S2, S3).

The following exclusion criteria were applied to all analyses: 1) missing lifestyle risk factors (physical activity or smoking status), 2) diagnosis of all-cause dementia before completion of the last Oxford WebQ, 3) missing socioeconomic factors (Townsend deprivation index, total household income, ethnic background, highest qualification, or overall health rating), 4) missing data of the physical exam (body mass index (BMI), systolic blood pressure (SBP)), 5) pre-existing malabsorption, 6) history of diabetes mellitus, and 7) implausible energy or carbohydrate intake, i.e., 0 kJ/d intake on at least one occasion, being in the upper 0.1% of total energy and/or carbohydrate consumption or total energy intake < 1.1 × basal metabolic rate—500 kcal (under-reporting) or > 2.5 × basal metabolic rate + 500 kcal (over-reporting). Basal metabolic rate was defined according to the Oxford equation [22].

An overview of all participants removed due to exclusion criteria is presented in Additional file 1: Fig. S2. A total of 186,622 participants were included in the present study. Written informed consent was obtained from all participants at baseline and ethical approval for the UK Biobank study was granted by the North West Multicentre Research Ethics Committee [20].

### Exposure assessment

To obtain detailed dietary information which includes the consumption of 206 food items and 32 beverages, a web-based 24 h dietary recall (Oxford WebQ) was completed [23]. The Oxford WebQ was specifically developed for use in large population studies [21]. The Oxford WebQ has recently been validated against accelerometry-estimated energy expenditure and biomarkers of total sugar intake, and performed well compared to traditional 24 h interviewer-led dietary recalls especially when at least two questionnaires were completed [24]. Based on the Oxford WebQ data, intake of sugar and sugar subtypes from beverages and solids was calculated with a methodology described in two previous reports from our group [18, 19]. In brief, soda/fruit drinks, pure juice, milk-based drinks, and sugar added to tea/coffee were defined as sugary beverages whereas treats, breakfast cereals, toppings, and sauces as subtypes of sugary solids. Portion sizes for all Oxford WebQ food items were taken from the UK Food Standards Agency [25] and product labels. In the Oxford WebQ, participants reported the number of standard servings consumed of specific food items.

The intake (g/d) of the specific sugar subtype was calculated by multiplying the reported consumption frequency of each food item by the estimated content of this sugar subtype in that item in one serving. To calculate sugar subtype intake in % total energy (%E), the intake in g/d was multiplied with 17 kJ/g * 100% / total energy in kJ/d according to Willett and co-workers [26]. The difference between total sugars and FS equals intrinsic sugar.

For participants who completed more than one questionnaire, the mean %E intake of sugar subtypes was used for all primary and sensitivity analyses except when only the first completed Oxford WebQ was considered (Additional file 1 Fig. S12).

### Outcome assessment

The primary outcome was incident all-cause dementia (termed dementia throughout the manuscript) which was provided by UK Biobank as an algorithmically-defined outcome, i.e., date of all-cause dementia report (data field 42,018) [27]. Follow-up time was defined as the period from the first dietary assessment to the date of the first diagnosis of dementia, loss-to-follow-up, death, or censoring, whichever came first. The analyses were censored at the censoring date for the hospital admission data, i.e., 30th of September 2021 for England, 31th of July 2021 for Scotland, and 28th of February 2018 for Wales, depending on the participants’ origin.

### Statistical analyses

Data analysis was performed with R version 4.2.2 [28] as described recently [19].

In brief, the hazard ratios (HR) for incident dementia were assessed with Cox proportional hazard regression multivariate nutrient density models [26] including %E intake of sugar from different sources and energy intake as penalized cubic splines with their degrees of freedom set to 4. We adjusted the models further for energy intake (penalized cubic splines), age at completion of the first Oxford WebQ (split by quintiles), alcohol intake (< 1, 1 to < 8, 8 to < 16, ≥ 16 g/d), BMI (< 18.5, 18.5 to < 25, 25 to < 30, ≥ 30 kg/m^2^), ethnic background (White, group composed of Mixed, Asian, Black, Chinese, and other), general health status (poor, fair, good, excellent), highest qualification (none of the below, national exams at age 16 years, vocational qualifications or optional national exams at ages 17–18 years, professional, College or University), history of mental illness (yes, no), physical activity (metabolic equivalent of task (MET)-minutes per week derived from the Oxford WebQ; split by quintiles), SBP (split by quintiles), sex (female, male), smoking status (never, previous, occasional, current < 10, 10 to 14, 15 to 19, ≥ 20 cigarettes per day), total household income (< 18, 18 to < 31, 31 to < 52, 52 to < 100, ≥ 100 k£, unknown), and Townsend deprivation index (split by quintiles). If there were deviations from the abovementioned adjustments, this is indicated in the figure legends. The hazard proportionality was evaluated for each covariate using scaled Schoenfeld residuals. All covariates that significantly violated the proportionality hazard assumption after Holm-adjustment for multiple testing were stratified in the final models. In each analysis, the determination of the nadir of the estimated HR as a function of the intake of a sugar subtype in %E was restricted to the range from zero to the 99%-quantile. The HR was then rescaled to a nadir of 1 to simplify the presentation. HRs with pointwise 95% confidence intervals (CIs) are shown for all Cox proportional hazard regression models. The analysis of each penalized cubic spline is divided into p^lin^ for the linear and p^non−lin^ for the nonlinear effect, as recently described [29]. A *p*-value of < 0.05 was considered as statistically significant in all analyses. We performed no further interpretation of the HR-nadir or other individual HRs if both p^lin^ and p^non−lin^ were non-significant.

### Sensitivity analyses

To assess the robustness of the results, several sensitivity analyses were performed similarly as described in recent studies [18, 19, 30]: Reverse causation was accounted for by excluding participants who were diagnosed with dementia within two years after filling out their first Oxford WebQ (landmark analysis) (*n* = 186,580, n excluded = 24,367), who had lost weight unintentionally (*n* = 157,057, n excluded = 53,890), or had a history of cardiovascular disease (CVD) and cancer (*n* = 164,855, n excluded = 46,092). Participants who filled out only one questionnaire were removed from the analysis to address potential variation, i.e., lower reproducibility in sugar intake based on a single Oxford WebQ [24] (*n* = 115,480, n excluded = 95,467). To control for unrepresentative consumption data, participants who described their diet on the previous day as non-typical on at least one occasion were excluded (*n* = 125,313, n excluded = 85,634). Participants who followed a restricted diet due to health reasons, i.e., participants indicating their diet as being “low-calorie”, “lactose-free”, or “gluten-free” (*n* = 160,752, n excluded = 50,195) were excluded in another set of sensitivity analyses. In order to explore heightened dementia risk with increasing age, the analyses were re-done in the subgroup of participants ≥ 60 years (*n* = 90,571, n excluded = 120,376), as well as stratified for age below or above 60 years (*n* = 186,622, n excluded = 24,325).To focus on the nutrient intake closest to baseline assessment, analyses were repeated using only the first Oxford WebQ questionnaire (*n* = 186,622, n excluded = 24,325). To further control for residual confounding by dietary factors, a diet quality score combining five dietary components, i.e., fat, fruit, vegetables, red meat, and processed meat consumption, was included in the analysis as described in [30] (*n* = 184,271, n excluded = 26,676). To apply alternative measures for body composition, waist-to-hip ratio (WHR) and height were used instead of BMI (*n* = 186,580, n excluded = 24,367). In order to include as many participants as possible in the analysis, two additional sensitivity analyses were carried out: Firstly, all missing values for any of the covariates were included as novel “unknown” category. For example, if a BMI value was not present, the participant was assigned the BMI category “unknown” and included in the analysis (*n* = 190,205, n excluded = 20,742). Secondly, only a minimal set of exclusion criteria was applied in addition to creating the novel “unknown” category as described above. Thus, participants were only excluded if dementia was pre-existent (*n* = 80) or no energy intake was reported (*n* = 46) (*n* = 210,821, n excluded = 126).

## Results

### Characteristics of UK Biobank participants

Table 1 illustrates the characteristics of the study population in total and in subgroups of FS intake defined by quintiles. Mean (standard deviation (SD)) age of the study cohort at completion of the first Oxford WebQ was 58 (8) years with 57.3% of participants being female. Time of follow-up was 10.6 (1.1) years, i.e., 2.0 million person-years, with a total of 1498 incident dementia cases of which 730 occurred in females and 768 in males.

**Table 1:** Characteristics of the UK Biobank cohort^*^

**Parameters**		Total cohort (*n* = 186,622)	**FS intake (%E) split by quintiles**				
			0.0 to 6.8 (*n* = 37,325)	6.8 to 9.5 (*n* = 37,324)	9.5 to 12.1 (*n* = 37,324)	12.1 to 15.5 (*n* = 37,324)	15.5 to 77.5 (*n* = 37,325)
**Characteristics**							
Age at completion of first Oxford WebQ (years)		58 (8)	58 (8)	58 (8)	58 (8)	58 (8)	57 (8)
BMI (kg/m^2^)		26.6 (4.3)	26.8 (4.4)	26.6 (4.3)	26.4 (4.2)	26.4 (4.3)	26.6 (4.4)
Ethnic background							
- White		179,879 (96.4)	36,088 (96.7)	36,251 (97.1)	36,162 (96.9)	36,101 (96.7)	35,277 (94.5)
- Mixed, Asian, Black, Chinese, and other		6,743 (3.6)	1,237 (3.3)	1,073 (2.9)	1,162 (3.1)	1,223 (3.3)	2,048 (5.5)
General health status							
- Poor		4,515 (2.4)	814 (2.2)	697 (1.9)	725 (1.9)	873 (2.3)	1,406 (3.8)
- Fair		29,894 (16.0)	5,985 (16.0)	5,544 (14.9)	5,499 (14.7)	5,877 (15.7)	6,989 (18.7)
- Good		113,190 (60.7)	22,613 (60.6)	22,707 (60.9)	23,043 (61.7)	22,838 (61.2)	21,989 (58.9)
- Excellent		39,023 (20.9)	7,913 (21.2)	8,376 (22.4)	8,057 (21.6)	7,736 (20.7)	6,941 (18.6)
Highest qualification							
- None of the below		14,984 (8.0)	3,307 (8.9)	2,788 (7.5)	2,800 (7.5)	2,818 (7.6)	3,271 (8.8)
- National exams at age 16 years		28,062 (15.0)	5,699 (15.3)	5,352 (14.3)	5,450 (14.6)	5,509 (14.8)	6,052 (16.2)
- Vocational qualifications or optional national exams at ages 17–18 years		33,058 (17.7)	6,771 (18.1)	6,440 (17.3)	6,377 (17.1)	6,381 (17.1)	7,089 (19.0)
- Professional		28,977 (15.5)	5,543 (14.9)	5,625 (15.1)	5,930 (15.9)	5,934 (15.9)	5,945 (15.9)
- College or University		81,541 (43.7)	16,005 (42.9)	17,119 (45.9)	16,767 (44.9)	16,682 (44.7)	14,968 (40.1)
History of mental illnesses	12,278 (6.6)		2,413 (6.5)	2,288 (6.1)	2,222 (6.0)	2,409 (6.5)	2,946 (7.9)
Physical activity (MET-min/week)		4,130 (2,651)	4,069 (2,663)	4,112 (2,572)	4,133 (2,559)	4,142 (2,598)	4,194 (2,852)
SBP (mmHg)		139 (19)	139 (20)	139 (19)	139 (19)	138 (19)	138 (19)
Sex – female		106,834 (57.3)	21,447 (57.5)	21,731 (58.2)	21,512 (57.6)	21,380 (57.3)	20,764 (55.6)
Smoking status							
- Never		107,357 (57.5)	19,187 (51.4)	20,906 (56.0)	21,870 (58.6)	22,713 (60.9)	22,681 (60.8)
- Previous		65,874 (35.3)	15,128 (40.5)	13,972 (37.4)	13,146 (35.2)	12,165 (32.6)	11,463 (30.7)
- Occasional		4,461 (2.4)	1,065 (2.9)	912 (2.4)	849 (2.3)	810 (2.2)	825 (2.2)
- Current < 10 cigarettes per day		2,313 (1.2)	491 (1.3)	415 (1.1)	385 (1.0)	476 (1.3)	546 (1.5)
- Current 10 to 14 cigarettes per day		2,011 (1.1)	410 (1.1)	349 (0.9)	332 (0.9)	361 (1.0)	559 (1.5)
- Current 15 to 19 cigarettes per day		1,787 (1.0)	376 (1.0)	302 (0.8)	294 (0.8)	323 (0.9)	492 (1.3)
- Current ≥ 20 cigarettes per day		2,819 (1.5)	668 (1.8)	468 (1.3)	448 (1.2)	476 (1.3)	759 (2.0)
Total household income per year (k£)							
- < 18		24,987 (13.4)	4,730 (12.7)	4,582 (12.3)	4,692 (12.6)	5,101 (13.7)	5,882 (15.8)
- 18 to < 31		40,795 (21.9)	7,767 (20.8)	7,956 (21.3)	8,176 (21.9)	8,333 (22.3)	8,563 (22.9)
- 31 to < 52		48,423 (26.0)	9,591 (25.7)	9,806 (26.3)	9,779 (26.2)	9,669 (25.9)	9,578 (25.7)
- 52 to < 100		41,708 (22.4)	9,877 (23.8)	8,751 (23.4)	8,469 (22.7)	8,179 (21.9)	7,432 (19.9)
- ≥ 100		12,437 (6.7)	2,857 (7.7)	2,735 (7.3)	2,537 (6.8)	2,345 (6.3)	1,963 (5.3)
- Unknown		18,272 (9.8)	3,503 (9.4)	3,494 (9.4)	3,671 (9.8)	3,697 (9.9)	3,907 (10.5)
Townsend deprivation index	-1.6 (2.8)		-1.5 (2.9)	-1.7 (2.8)	-1.7 (2.8)	-1.8 (2.8)	-1.5 (2.9)
**Dietary sugar subtype intake in %E**							
Carbohydrates		48.6 (7.8)	44.4 (8.9)	46.8 (7.3)	48.4 (6.7)	50.1 (6.3)	53.5 (6.6)
Total sugars		24.4 (7.2)	18.8 (6.6)	21.8 (5.6)	23.9 (5.3)	26.2 (5.2)	31.1 (6.3)
Intrinsic sugars		13.0 (5.7)	14.4 (6.4)	13.6 (5.6)	13.1 (5.3)	12.5 (5.1)	11.4 (5.3)
FS		11.4 (5.6)	4.5 (1.7)	8.2 (0.8)	10.8 (0.7)	13.7 (1.0)	19.7 (4.3)
FS beverages		4.7 (4.7)	1.1 (1.4)	2.6 (2.1)	3.9 (2.5)	5.6 (3.1)	10.5 (5.9)
- Soda/fruit drinks		1.6 (3.3)	0.2 (0.6)	0.5 (1.2)	0.9 (1.7)	1.6 (2.4)	4.6 (5.4)
- Juice		2.1 (2.8)	0.6 (1.2)	1.4 (1.8)	2.1 (2.2)	2.7 (2.6)	3.8 (4.0)
- Milk-based drinks		0.3 (0.9)	0.1 (0.5)	0.2 (0.7)	0.3 (0.9)	0.4 (1.0)	0.6 (1.3)
- Tea/coffee		0.6 (1.6)	0.2 (0.6)	0.3 (1.0)	0.5 (1.2)	0.7 (1.6)	1.4 (2.6)
FS solids		6.6 (3.5)	3.4 (1.8)	5.6 (2.1)	6.9 (2.5)	8.1 (3.0)	9.2 (4.1)
- Treats		4.4 (3.0)	2.1 (1.6)	3.6 (2.0)	4.5 (2.4)	5.3 (2.8)	6.3 (3.8)
- Cereals		0.5 (0.8)	0.3 (0.6)	0.5 (0.7)	0.5 (0.8)	0.6 (0.8)	0.6 (0.9)
- Toppings		1.2 (1.6)	0.4 (0.9)	0.9 (1.4)	1.3 (1.6)	1.6 (1.8)	1.7 (2.0)
- Sauces		0.3 (0.4)	0.2 (0.4)	0.3 (0.4)	0.3 (0.4)	0.3 (0.4)	0.3 (0.4)
**Other nutrients of interest**							
Alcohol (g/d)		17.3 (22.1)	26.1 (28.5)	20.2 (22.9)	16.7 (19.7)	13.7 (17.7)	9.7 (15.5)
Fat (g/d)		79.3 (28.0)	73.9 (27.4)	79.6 (27.5)	81.8 (27.8)	82.3 (27.9)	78.9 (28.5)
Protein (g/d)		74.7 (20.8)	76.5 (22.6)	76.7 (20.6)	76.0 (19.9)	74.4 (19.6)	70.1 (20.1)
Fibre (g/d)		19.1 (7.0)	19.3 (7.7)	19.8 (7.1)	19.6 (6.8)	19.1 (6.6)	17.5 (6.8)
Energy (kJ/d)		9,090 (2,309)	8,532 (2,249)	9,002 (2,236)	9,223 (2,255)	9,347 (2,290)	9,347 (2,412)
**Number of Oxford WebQ**		2.2 (1.2)	2.0 (1.1)	2.3 (1.2)	2.4 (1.2)	2.3 (1.2)	2.0 (1.1)

*Abbreviations: BMI* Body mass index, *FS* Free sugars, *g/d* Grams per day, *kg/m*^*2*^ Kilogram per square meter, *kJ* Kilojoules, *MET* Metabolic equivalent of task, *mmHg* Millimetres of mercury, *%E* Percentage total energy, *SBP* Systolic blood pressure, *SD* Standard deviation

^*^Categorical variables are summarized as frequencies (percentages) and continuous variables as mean (SD)

### FS versus intrinsic sugars

**Fig. 1:**
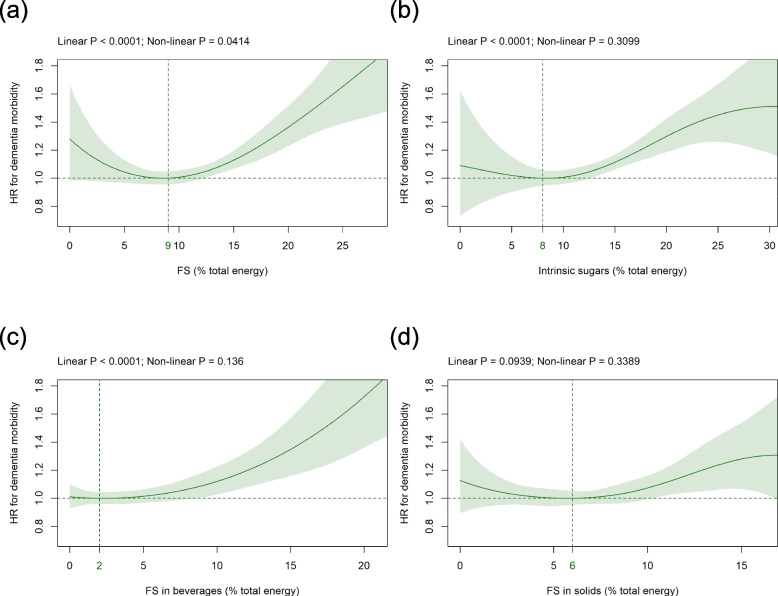
Association of **a** FS, **b** intrinsic sugars, **c** FS in beverages, and **d** FS in solids intake (all %E) with dementia risk. Models are adjusted for energy intake (penalized cubic splines), age (split by quintiles), alcohol intake (< 1, 1 to < 8, 8 to < 16, ≥ 16 g/d), BMI (< 18.5, 18.5 to < 25, 25 to < 30, ≥ 30 kg/m^2^), ethnic background (White, group composed of Mixed, Asian, Black, Chinese, and other), general health status (poor, fair, good, excellent), highest qualification (none of the below, national exams at age 16 years, vocational qualifications or optional national exams at ages 17–18 years, professional, College or University), history of mental illness (yes, no), physical activity (metabolic equivalent of task (MET)-minutes per week derived from the Oxford WebQ; split by quintiles), SBP (split by quintiles), sex (female, male), smoking status (never, previous, current occasional, current < 10, 10 to 14, 15 to 19, ≥ 20 cigarettes per day), total household income (< 18, 18 to < 31, 31 to < 52, 52 to < 100, ≥ 100 k£, unknown), and Townsend deprivation index (split by quintiles). Covariates not fulfilling the proportional hazard assumption are stratified. The HR-nadir is indicated by the vertical line. *Abbreviations: BMI* Body mass index, *FS* Free sugars, *HR* Hazard ratio, *kg/m*^*2*^ Kilogram per square meter, *MET* Metabolic equivalent of task, *%E* Percentage total energy, *SBP* Systolic blood pressure

Mean (SD) consumption of FS and intrinsic sugars was 11.4 (5.6) %E and 13.0 (5.7) %E, respectively (Table 1). FS intake was significantly associated with the HR for dementia in a J-shaped fashion (Fig. 1a). The HR-nadir was found at 9%E FS (Fig. 1a). Compared to intake at the nadir, the HR (CI) increased to 1.28 (0.98 to 1.67) and 1.36 (1.23 to 1.51), at 0 and 20%E, respectively (Fig. 1a). All sensitivity analyses showed a significant association between FS and HR for dementia (Additional file 1 Fig. S4a to 16a) which changed from a J-shape to a more linear shape if participants who filled out only one questionnaire were removed from the analysis (Additional file 1 Fig. S7a). The intake of intrinsic sugars was also significantly related to dementia risk in a J-shaped fashion and the HR-nadir was observed at 8%E (Fig. 1b). Compared to the intake at the nadir, the HR (CI) increased to 1.30 (1.19 to 1.42) at 20%E (Fig. 1b). Intrinsic sugars remained significantly associated with dementia risk in all sensitivity analyses (Additional file 1 Fig. S4b to S16b). The relation between intrinsic sugars and dementia risk changed from a J-shape to a more linear shape in three sensitivity analyses (Additional file 1 Fig. S7b, S9b, S12b).

### FS in beverages versus FS in solids

Mean (SD) intake of FS in beverages and FS in solids was 4.7 (4.7) %E and 6.6 (3.5) %E, respectively (Table 1). Dementia risk was significantly associated with FS in beverage intake in an ascending approximately linear way (Fig. 1c). The HR-nadir was observed at 2%E FS and the HRs (CIs) increased to 1.12 (1.02 to 1.22) and 1.72 (1.36 to 2.19) at 10%E and 20%E, respectively (Fig. 1c). The relation between incident dementia and FS in beverages remained similar in all sensitivity analyses (Additional file 1 Fig. S4c to S16c). The relation between FS in solids and dementia risk was not significant in the primary analysis (Fig. 1d) but in four out of the 13 sensitivity analyses (Additional file 1 Fig. S6d, S7d, S11d, S16d), changing towards a more linear shape in Additional file 1 Fig. S7d.

### FS in beverage subtypes

**Fig. 2:**
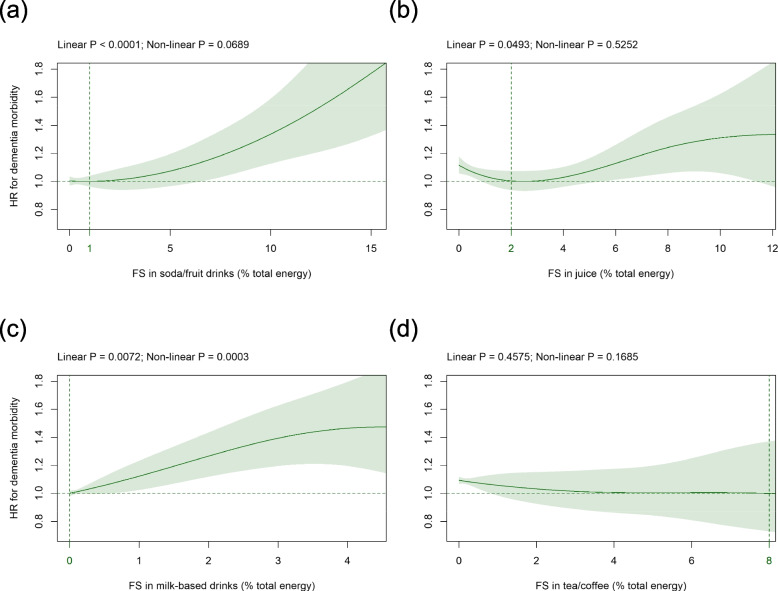
Association of FS in **a** soda/fruit drinks,** b** juice, **c** milk-based drinks, and **d** tea/coffee (all %E) with dementia risk. Models are adjusted and presented as indicated in Fig. 1. *Abbreviations: %E* Percentage total energy

Mean (SD) intake of FS in beverage subtypes was as follows: soda/fruit drinks 1.6 (3.3), juice 2.1 (2.8), milk-based drinks 0.3 (0.9), and tea/coffee 0.6 (1.6) %E (Table 1). FS in soda/fruit drinks were significantly approximately linearly ascending associated with dementia risk with the HR-nadir found at 1%E and HR (CI) of 1.34 (1.13 to 1.59) at 10%E FS (Fig. 2a). FS in juice were significantly related with HR for dementia in a linear manner with the HR-nadir observed at 2%E and HRs (CIs) of 1.12 (1.06 to 1.18) and 1.31 (1.06 to 1.62) at 0%E and 10%E FS, respectively (Fig. 2b). FS in milk-based drinks were significantly approximately linearly ascending associated with dementia risk with the HR-nadir detected at 0%E and increased HR (CI) of 1.39 (1.19 to 1.63) at 3%E FS but with a flattening trend at consumption levels above 4%E (Fig. 2c). These findings were robust in all sensitivity analyses except for FS in juice which only remained significantly associated with dementia in four out the 13 sensitivity analyses (Additional file 1 Fig. S4f, S14f, S15f, S16f). FS in tea/coffee were not significantly related to incident dementia (Fig. 2d) except for two sensitivity analyses (Additional file 1 Fig. S5h, S7h).

### FS in solids subtypes

**Fig. 3:**
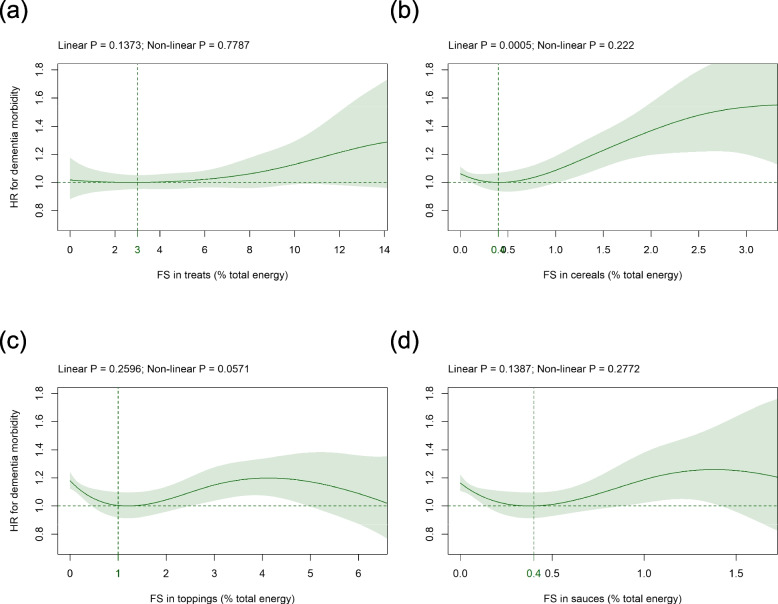
Association of FS in **a** treats, **b** cereals,** c** toppings, and **d** sauces (all %E) with dementia risk. Models are adjusted and presented as indicated in Fig. 1. *Abbreviations: %E* Percentage total energy

Mean (SD) intake of FS in solids subtypes was as follows: treats 4.4 (3.0), cereals 0.5 (0.8), toppings 1.2 (1.6), and sauces 0.3 (0.4) %E (Table 1). Concerning the solids subtypes studied, FS in treats were not significantly associated with dementia risk in the primary (Fig. 3a) and all sensitivity analyses with the exception of three sensitivity analyses (Additional file 1 Fig. S6h, S7h, S16h). FS in cereals were significantly related to incident dementia in a linear to sigmoid way in the primary analysis with the HR-nadir at 0.4%E (Fig. 3b) and all sensitivity analyses (Additional file 1 Fig. S4h to S16h). FS in toppings were not significantly related to dementia risk but became significant in three sensitivity analyses (Additional file 1 Fig. S6k, S10k, S15k). FS in sauces did not show a significant association with dementia incidence with the exception of one sensitivity analysis (Additional file 1 Fig. S9l).

## Discussion

### Principal findings

In the current study, it is elucidated for the first time in a large prospective cohort how FS from all major liquid and solid sources and their subtypes are associated with dementia risk. Furthermore, we are the first to use penalized cubic splines to allow for non-linear predictor effects.

Overall FS intake is significantly associated with dementia risk in a J-shaped fashion with the HR-nadir found at 9%E. Similarly, intrinsic sugars are significantly related with dementia risk in a J-shaped fashion with the HR-nadir at 8%E. FS in beverages are significantly related to dementia risk in an ascending approximately linear way, whereas no association is found for FS in solids. Within the beverage subtypes, FS in soda/fruit drinks, milk-based drinks and to a lesser extent in juice are significantly and positively related to dementia risk, whereas no association is found for FS in tea/coffee. Our results highlight that the associations between FS and dementia risk depend on FS source.

### Comparison with other studies

The mean FS consumption of UK Biobank participants in the present study of 11.4%E is slightly higher as compared to the median daily intake of 9%E in a representative UK population sample from the National Diet and Nutrition Survey Rolling Programme 2014–2016 [31].

To the best of our knowledge, the current study is the first to assess the association between FS from all sources and incident dementia. A cross-sectional study by Ye and co-workers in 737 participants shows convincingly that increasing added sugar intake is inversely related to cognitive function when comparing the first quintile to all other quintiles [32].

The present analysis is by far the largest study indicating a dose-dependency of FS in beverages intake and dementia risk. The majority of studies so far has focused exclusively on soda/fruit drinks with participant numbers ranging from 1384 to 22,564 and showing a positive association with dementia [33–36], similar to our present findings. Only one study in 16,948 participants does not find a significant association between soda/fruit drinks and cognitive impairment [37]. Differences in study results may be explained by relatively low consumption levels, different outcome measurements such as cognitive impairment, incident cases of Alzheimer’s disease, and cognitive function, as well as varying model adjustments. Taking these and the current findings into consideration, a positive link between FS in soda/fruit drinks and incident dementia is found in the majority of studies.

In our study, intake of FS in juice is related to incident dementia in a linear manner with the HR-nadir at 2%E. Two studies show either an inverse association of juice intake > 3 times/week with HR for incident Alzheimer’s disease compared to intake less often than weekly [38] or no association with cognitive function [39].

These data suggest that low to moderate juice intake is not linked to increased dementia risk and might even be a protective factor for the development of the disease. In contrast to FS in soda/fruit drinks and juice, no study so far has assessed the association between FS in milk-based sugary drinks, as well as tea/coffee, and incident dementia. FS in milk-based drinks show a positive association with dementia risk similar to FS in soda/fruit drinks while there is no relation for FS in tea/coffee.

Our study is the first to analyse the association of FS in solid foods with dementia risk and no significant link is found. In agreement with our findings, FS in solids are not associated with incident dementia in a cross-sectional study of 737 middle-aged Puerto Ricans [32].

To the best of our knowledge, the association of intrinsic sugars with dementia risk is characterized for the first time in the current study. The largest amounts of intrinsic sugars can be found in fruits and vegetables, as well as in milk with naturally present lactose and galactose [16]. Our current results of a J-shaped association between intrinsic sugars and dementia risk which even changes to a more linear shape in some sensitivity analyses are unexpected since fruits, vegetables, and dairy products have been linked with a decreased risk of dementia in several [40–43] but not all [39] studies. It is interesting to note in this context that in two recent studies from our group in the same study population intrinsic sugars are not significantly related to all-cause mortality and incident depression [18, 19]. More large-scale studies on the effect of intrinsic sugars as such on dementia risk are warranted.

### Strengths and limitations

Strengths of the current study include a large sample size, the prospective cohort design, a thorough characterization of participants, a mean follow-up > 10 years, a wide range of sugar subtype intake, as well as analyses with penalized cubic splines to allow non-linear predictor effects. A recent study confirmed the applicability of the UK Biobank classification for dementia in prospective studies to identify all-cause dementia [44]. Limitations of the present study include residual confounding, which might lead to bias because estimated associations between outcome and exposure can be affected by unmeasured confounding factors. Moreover, measurement errors in the assessment of the exposure variables, and potential confounders might have occurred possibly altering the results. Since self-reported dietary assessments are vulnerable to reporting errors, only one assessment might not give a reliable insight into usual dietary habits. It is important to note in this context that the association between sugar subtypes and dementia risk changes towards a more linear association especially for intrinsic sugars and FS in solids if the analyses are restricted to participants who have completed, at a minimum, two questionnaires. The increased dementia risk in the no/low intake group might also represent a certain group of people who made dietary changes for or have a restricted diet for health reasons. This association is similar to the consistent J-shaped associations observed for alcohol intake and health outcomes, with various sources of bias and confounding potentially driving these associations [45]. Interestingly, the association of intrinsic sugars with dementia risk changes from a J-shape to a more linear shape if participants with a restricted diet due to health reasons are excluded. Reverse causality might be possible, i.e., even before dementia is diagnosed, the disease might influence dietary habits. However, results remain virtually unchanged in landmark analyses excluding all participants who have developed dementia within two years after completion of their first Oxford WebQ. Furthermore, a “healthy volunteer” selection bias is possible since it introduces a lack of representativeness into the cohort [46]. However, this is not necessarily a limitation since the size of the UK Biobank and the heterogeneity of exposure measures still allow the assessment of exposure-disease relationships [46].

## Conclusions

A linear-shaped association between sugar subtype intake and dementia risk is most consistently found for FS in beverages and more specifically for FS in soda/fruit drinks, as well as in milk-based drinks. The association between intrinsic sugars and dementia risk which becomes linear in some sensitivity analyses warrants further assessment.

Further prospective studies on sugar subtype intake in relation to other disease states including CVD and cancer are necessary to provide an even more definitive conclusion.

### Supplementary Information

**Additional file 1: Figure S1.** Sugar sources relevant to the present study. **Figure S2.** Flowchart of participant selection. **Figure S3.** Venn diagram depicting number of participants excluded by seven exclusion criteria. **Figure S4.** Landmark analysis. **Figure S5.** Unintentional weight loss removed. **Figure S6.** Participants with history of CVD and cancer removed. **Figure S7.** Participants with only one completed Oxford WebQ removed. **Figure S8.** Non-typical diet removed. **Figure S9.** Special diet removed. **Figure S10.** Restricted to participants with age ≥ 60 years. **Figure S11.** Stratified by age (< 60 and ≥ 60 years). **Figure S12.** First Oxford WebQ only. **Figure S13.** Adjustment for diet quality score. **Figure S14.** Adjustment for WHR and height instead of BMI. **Figure S15.** Missing values of covariates recoded as “unknown” category. **Figure S16.** Only minimal set of exclusion criteria applied.

## Data Availability

The data that support the findings of this study are available from UK Biobank but restrictions apply to the availability of these data, which were used under license for Application 53,438, and, therefore, are not publicly available. Bona fide researchers can apply to use the UK Biobank dataset by registering and applying at https://www.ukbiobank.ac.uk/enable-your-research/register.

## Abbreviations

%E: Percentage total energy
BMI: Body mass index
CI: Confidence interval
CVD: Cardiovascular disease
FS: Free sugars
g/d: Grams per day
HR: Hazard ratio
kJ: Kilojoules
MET: Metabolic equivalent of task
NHS: National Health Service England
SBP: Systolic blood pressure
SD: Standard deviation
WHO: World Health Organization
WHR: Waist-to-hip-ratio
