# Supplementary material for: Association of sugar intake from different sources with incident dementia in the prospective cohort of UK Biobank participants
Source: Nutr J. 2023 Sep 4;22:42. doi: 10.1186/s12937-023-00871-8 (PMC10476309; doi:10.1186/s12937-023-00871-8)
Supplement: Supplementary file 1 — Additional file 1: Figure S1. Sugar sources relevant to the present study. Figure S2. Flowchart of participant selection. Figure S3. Venn diagram depicting number of participants excluded by seven exclusion criteria. Figure S4. Landmark analysis. Figure S5. Unintentional weight loss removed. Figure S6. Participants with history of CVD and cancer removed. Figure S7. Participants with only one completed Oxford WebQ removed. Figure S8. Non-typical diet removed. Figure S9. Special diet removed. Figure S10. Restricted to participants with age ≥ 60 years. Figure S11. Stratified by age (< 60 and ≥ 60 years). Figure S12. First Oxford WebQ only. Figure S13. Adjustment for diet quality score. Figure S14. Adjustment for WHR and height instead of BMI. Figure S15. Missing values of covariates recoded as “unknown” category. Figure S16. Only minimal set of exclusion criteria applied. [file 12937_2023_871_MOESM1_ESM.pdf]

Supplementary Information

Nutrition Journal

Association of sugar intake from different sources with incident dementia in the prospective cohort of UK Biobank participants

Sylva M Schaefer[1#*]; Anna Kaiser[1#]; Gerrit Eichner[2§]; Mathias Fasshauer[1,3§]

1. Institute of Nutritional Science, Justus-Liebig University of Giessen, Giessen, Germany.
2. Mathematical Institute, Justus-Liebig University of Giessen, Giessen, Germany.
3. Center for Sustainable Food Systems, Justus-Liebig University of Giessen, Giessen, Germany.

[#]SMS and AK contributed equally to this work and are joint first authors.

[§]GE and MF contributed equally to this work and are joint senior authors.

*Corresponding author. Mailing address: Goethestr. 55, 35390 Giessen, Germany. Phone: +49 641 9939067. E-mail: sylva.schaefer@ernaehrung.uni-giessen.de

<u>**Additional File 1: Figures**</u>

Figure S1

Sugar sources relevant to the present study.

Figure S2

Flowchart of participant selection

Figure S3

Venn diagram depicting number of participants excluded by seven exclusion criteria: 1) missing lifestyle risk factors (physical activity or smoking status), 2) diagnosis of all-cause dementia before completion of the last Oxford WebQ, 3) missing socioeconomic factors (Townsend deprivation index, total household income, ethnic background, highest qualification, or overall health rating), 4) missing data of the physical exam (body mass index (BMI), systolic blood pressure (SBP)), 5) pre-existing malabsorption, 6) history of diabetes mellitus, and 7) implausible energy or carbohydrate intake, i.e., 0 kJ/d intake on at least one occasion, being in the upper 0.1 % of total energy and/or carbohydrate consumption or total energy intake <1.1 × basal metabolic rate - 500 kcal (under-reporting) or >2.5 × basal metabolic rate + 500 kcal (over-reporting) resulting in a study population of 186,622 participants.

Figure S4

Landmark analysis

Association of **a** FS, **b** intrinsic sugars, as well as FS in **c** beverages, **d** solids, **e** soda/fruit drinks, **f** juice, **g** milk-based drinks, **h** tea/coffee, **i** treats, **j** cereals, **k** toppings, and **l** sauces (all %E) with dementia risk (landmark analysis; n=186,580; number of cases=1,456). Models are adjusted for energy intake, age, alcohol intake, BMI, ethnic background, general health status, highest qualification, history of mental illnesses, physical activity, SBP, sex, smoking status, total household income, and Townsend deprivation index. Covariates not fulfilling the proportional hazard assumption are stratified. The nadir is indicated by the vertical line. *Abbreviations*: *BMI* Body mass index, *FS* Free sugars, *HR* Hazard ratio, *%E* Percentage total energy, *SBP* Systolic blood pressure

Figure S5

Unintentional weight loss removed

Association of **a** FS, **b** intrinsic sugars, as well as FS in **c** beverages, **d** solids, **e** soda/fruit drinks, **f** juice, **g** milk-based drinks, **h** tea/coffee, **i** treats, **j** cereals, **k** toppings, and **l** sauces (all %E) with dementia risk (unintentional weight loss removed; n=157,057; number of cases=1,223). Models are adjusted and presented as indicated in Additional file 1 Fig. S4. *Abbreviations*: *FS* Free sugars, *HR* Hazard ratio, *%E* Percentage total energy

Figure S6

Participants with history of CVD and cancer removed

Association of **a** FS, **b** intrinsic sugars, as well as FS in **c** beverages, **d** solids, **e** soda/fruit drinks, **f** juice, **g** milk-based drinks, **h** tea/coffee, **i** treats, **j** cereals, **k** toppings, and **l** sauces (all %E) with dementia risk (participants with history of CVD and cancer removed; n=164,855; number of cases=1,180). Models are adjusted and presented as indicated in Additional file 1 Fig. S4. *Abbreviations*: *FS* Free sugars, *HR* Hazard ratio, *%E* Percentage total energy

Figure S7

Participants with only one completed Oxford WebQ removed

Association of **a** FS, **b** intrinsic sugars, as well as FS in **c** beverages, **d** solids, **e** soda/fruit drinks, **f** juice, **g** milk-based drinks, **h** tea/coffee, **i** treats, **j** cereals, **k** toppings, and **l** sauces (all %E) with dementia risk (participants with only one completed Oxford WebQ removed; n=115,480; number of cases=776). Models are adjusted and presented as indicated in Additional file 1 Fig. S4. *Abbreviations*: *FS* Free sugars, *HR* Hazard ratio, *%E* Percentage total energy

Figure S8

Non-typical diet removed

Association of **a** FS, **b** intrinsic sugars, as well as FS in **c** beverages, **d** solids, **e** soda/fruit drinks, **f** juice, **g** milk-based drinks, **h** tea/coffee, **i** treats, **j** cereals, **k** toppings, and **l** sauces (all %E) with dementia risk (non-typical diet removed; n=125,313; number of cases=1,185). Models are adjusted and presented as

indicated in Additional file 1 Fig. S4. *Abbreviations*: *FS* Free sugars, *HR* Hazard ratio, *%E* Percentage total energy

Figure S9

Special diet removed

Association of **a** FS, **b** intrinsic sugars, as well as FS in **c** beverages, **d** solids, **e** soda/fruit drinks, **f** juice, **g** milk-based drinks, **h** tea/coffee, **i** treats, **j** cereals, **k** toppings, and **l** sauces (all %E) with dementia risk (special diet removed; n=160,752; number of cases=1,274). Models are adjusted and presented as indicated in Additional file 1 Fig. S4. *Abbreviations*: *FS* Free sugars, *HR* Hazard ratio, *%E* Percentage total energy

Figure S10

Restricted to participants with age ≥ 60 years

Association of **a** FS, **b** intrinsic sugars, as well as FS in **c** beverages, **d** solids, **e** soda/fruit drinks, **f** juice, **g** milk-based drinks, **h** tea/coffee, **i** treats, **j** cereals, **k** toppings, and **l** sauces (all %E) with dementia risk (restricted to participants with age ≥ 60 years; n=90,571; number of cases=1,361). Models are adjusted and presented as indicated in Additional file 1 Fig. S4. *Abbreviations*: *FS* Free sugars, *HR* Hazard ratio, *%E* Percentage total energy

Figure S11

Stratified by age (< 60 and ≥ 60 years)

Association of **a** FS, **b** intrinsic sugars, as well as FS in **c** beverages, **d** solids, **e** soda/fruit drinks, **f** juice, **g** milk-based drinks, **h** tea/coffee, **i** treats, **j** cereals, **k** toppings, and **l** sauces (all %E) with dementia risk (models were further stratified by age; n=186,622; number of cases=1,498). Models are adjusted and presented as indicated in Additional file 1 Fig. S4 except for the age covariate being replaced by a strata of age </≥ 60 years. *Abbreviations*: *FS* Free sugars, *HR* Hazard ratio, *%E* Percentage total energy

Figure S12

First Oxford WebQ only

Association of **a** FS, **b** intrinsic sugars, as well as FS in **c** beverages, **d** solids, **e** soda/fruit drinks, **f** juice, **g** milk-based drinks, **h** tea/coffee, **i** treats, **j** cereals, **k** toppings, and **l** sauces (all %E) with dementia risk (only the first Oxford WebQ was used for intake estimation; n=186,622; number of cases=1,498). Models are adjusted and presented as indicated in Additional file 1 Fig. S4. *Abbreviations*: *FS* Free sugars, *HR* Hazard ratio, *%E* Percentage total energy

Figure S13

Adjustment for diet quality score

Association of **a** FS, **b** intrinsic sugars, as well as FS in **c** beverages, **d** solids, **e** soda/fruit drinks, **f** juice, **g** milk-based drinks, **h** tea/coffee, **i** treats, **j** cereals, **k** toppings, and **l** sauces (all %E) with dementia risk (models were further adjusted for diet quality score; n=184,271; number of cases=1,457). Models are adjusted and presented as indicated in Additional file 1 Fig. S4. *Abbreviations*: *FS* Free sugars, *HR* Hazard ratio, *%E* Percentage total energy

Figure S14

Adjustment for WHR and height instead of BMI

Association of **a** FS, **b** intrinsic sugars, as well as FS in **c** beverages, **d** solids, **e** soda/fruit drinks, **f** juice, **g** milk-based drinks, **h** tea/coffee, **i** treats, **j** cereals, **k** toppings, and **l** sauces (all %E) with dementia risk (models were adjusted for WHR and height instead of BMI; n=186,580; number of cases=1,498). Models are adjusted and presented as indicated in Additional file 1 Fig. S4. *Abbreviations*: *FS* Free sugars, *HR* Hazard ratio, *%E* Percentage total energy

Figure S15

Missing values of covariates recoded as “unknown” category

Association of **a** FS, **b** intrinsic sugars, as well as FS in **c** beverages, **d** solids, **e** soda/fruit drinks, **f** juice, **g** milk-based drinks, **h** tea/coffee, **i** treats, **j** cereals, **k** toppings, and **l** sauces (all %E) with dementia risk (missing values of covariates recoded as “unknown” category; n=190,205; number of cases=1,546). Models are

adjusted and presented as indicated in Additional file 1 Fig. S4. *Abbreviations*: *FS* Free sugars, *HR* Hazard ratio, *%E* Percentage total energy

Figure S16

Only minimal set of exclusion criteria applied

Association of **a** FS, **b** intrinsic sugars, as well as FS in **c** beverages, **d** solids, **e** soda/fruit drinks, **f** juice, **g** milk-based drinks, **h** tea/coffee, **i** treats, **j** cereals, **k** toppings, and **l** sauces (all %E) with dementia risk (only minimal set of exclusion criteria applied; n=210,821; number of cases=1,859). Models are adjusted and presented as indicated in Additional file 1 Fig. S4. *Abbreviations*: *FS* Free sugars, *HR* Hazard ratio, *%E* Percentage total energy

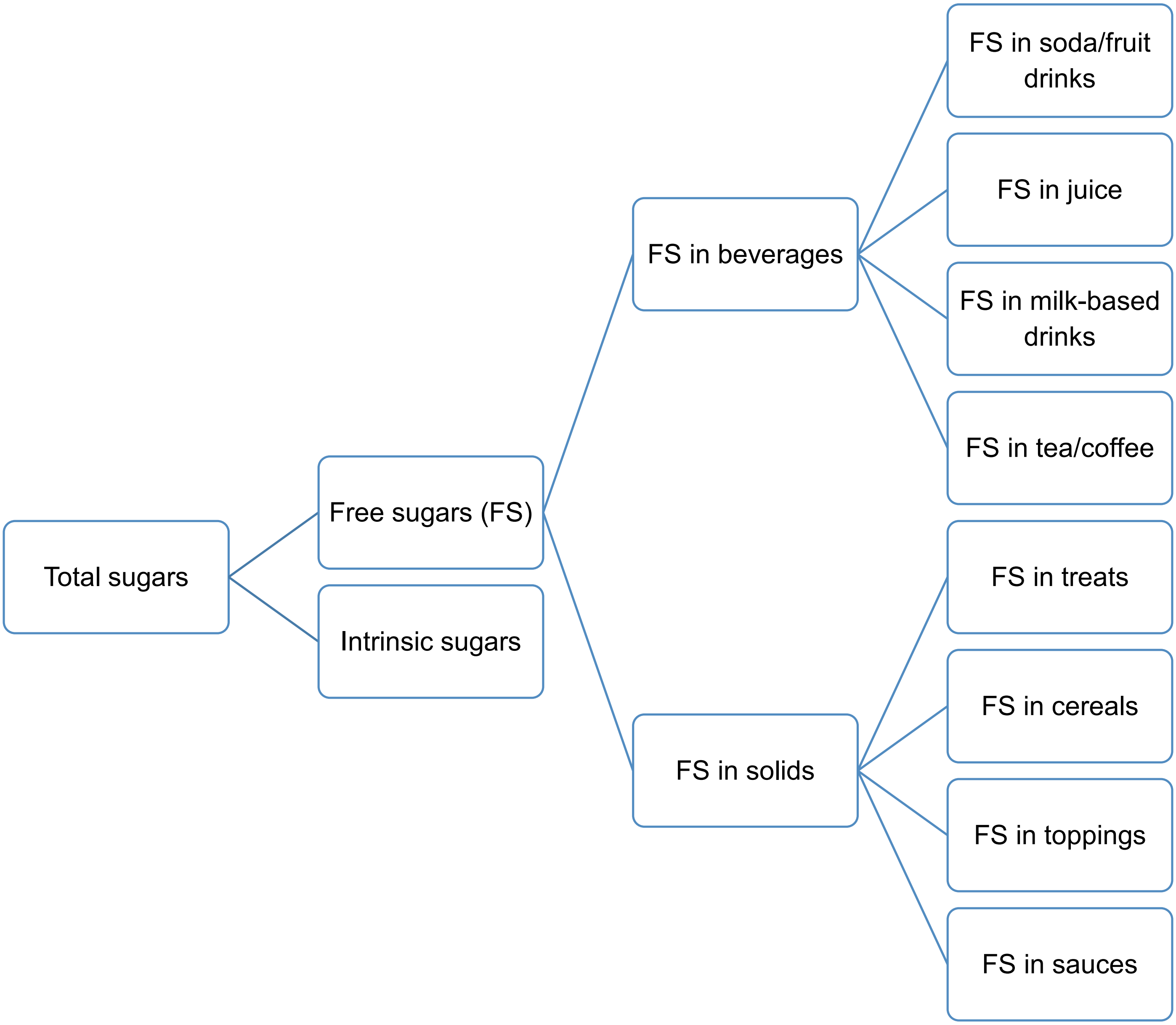
Total sugars
Free sugars (FS)
Intrinsic sugars
FS in beverages
FS in solids
FS in soda/fruit drinks
FS in juice
FS in milk-based drinks
FS in tea/coffee
FS in treats
FS in cereals
FS in toppings
FS in sauces

Figure S1

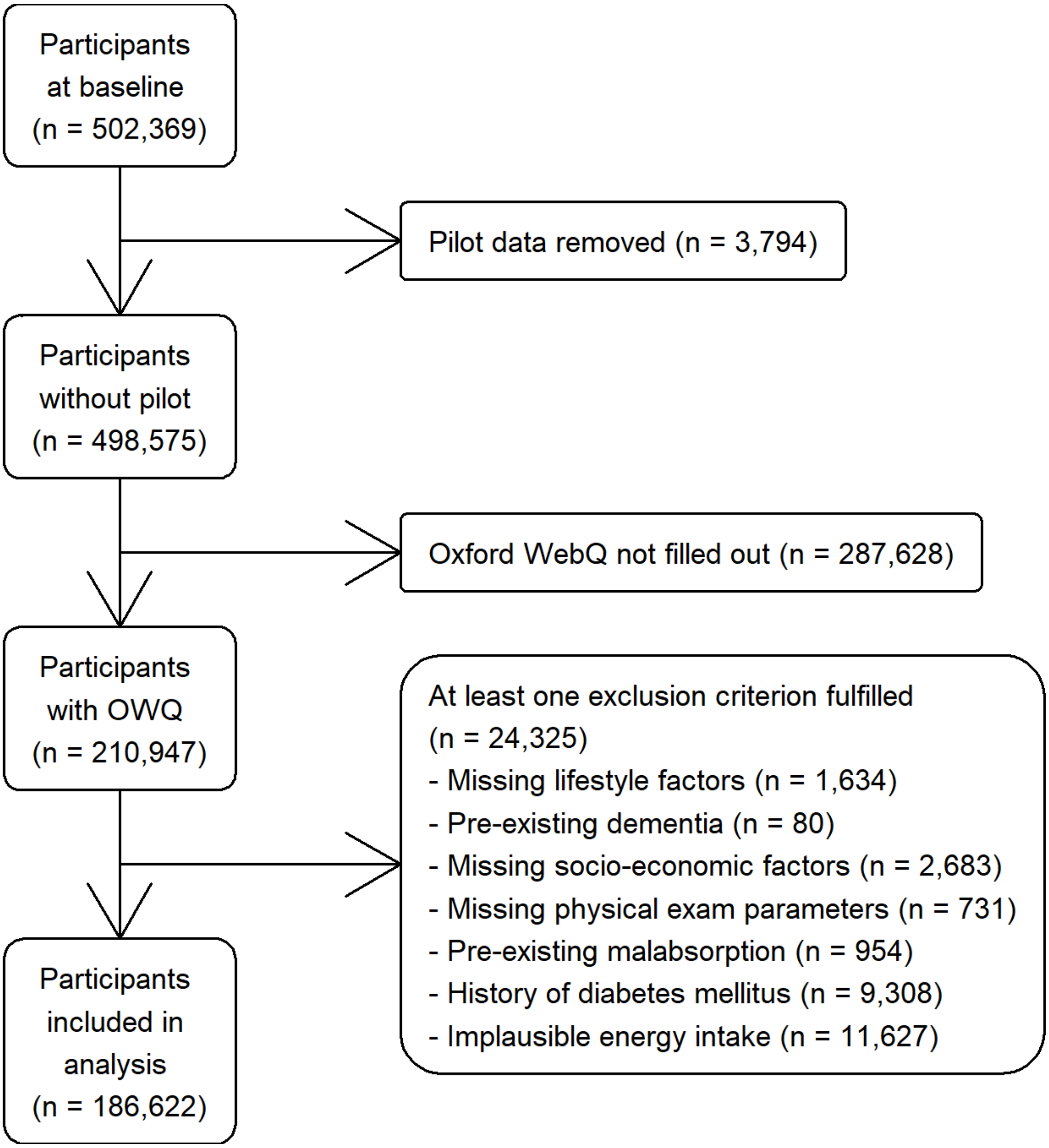
Participants at baseline (n = 502,369)
Pilot data removed (n = 3,794)
Participants without pilot (n = 498,575)
Oxford WebQ not filled out (n = 287,628)
Participants with OWQ (n = 210,947)
At least one exclusion criterion fulfilled (n = 24,325)
- Missing lifestyle factors (n = 1,634)
- Pre-existing dementia (n = 80)
- Missing socio-economic factors (n = 2,683)
- Missing physical exam parameters (n = 731)
- Pre-existing malabsorption (n = 954)
- History of diabetes mellitus (n = 9,308)
- Implausible energy intake (n = 11,627)
Participants included in analysis (n = 186,622)

Figure S2

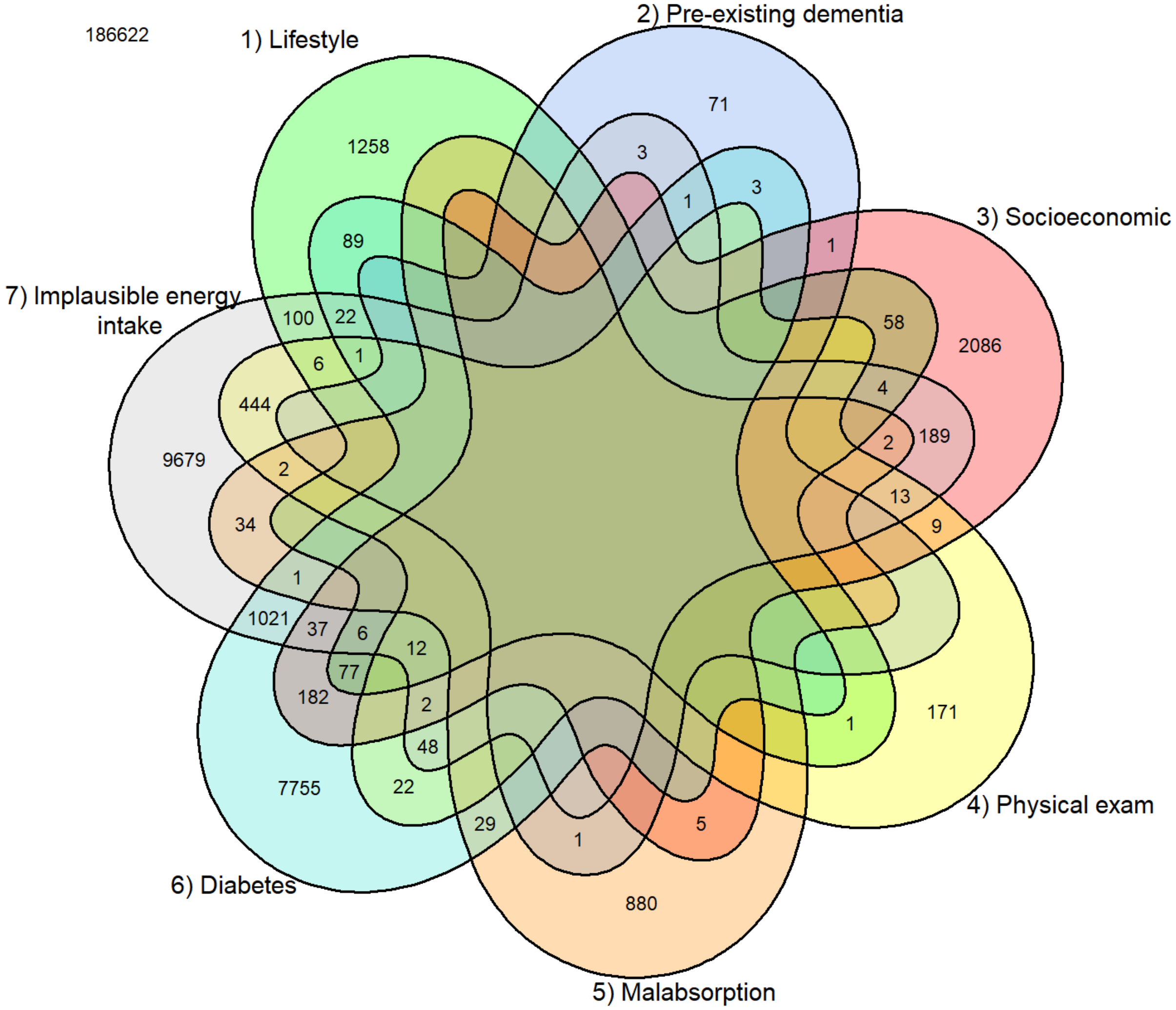
186622
1) Lifestyle
2) Pre-existing dementia
3) Socioeconomic
4) Physical exam
5) Malabsorption
6) Diabetes
7) Implausible energy intake
1258
71
2086
171
880
7755
9679
3
3
1
1
89
100
22
6
1
444
2
34
1
1021
37
6
12
77
182
2
48
22
29
1
5
1
58
4
189
2
13
9

Figure S3

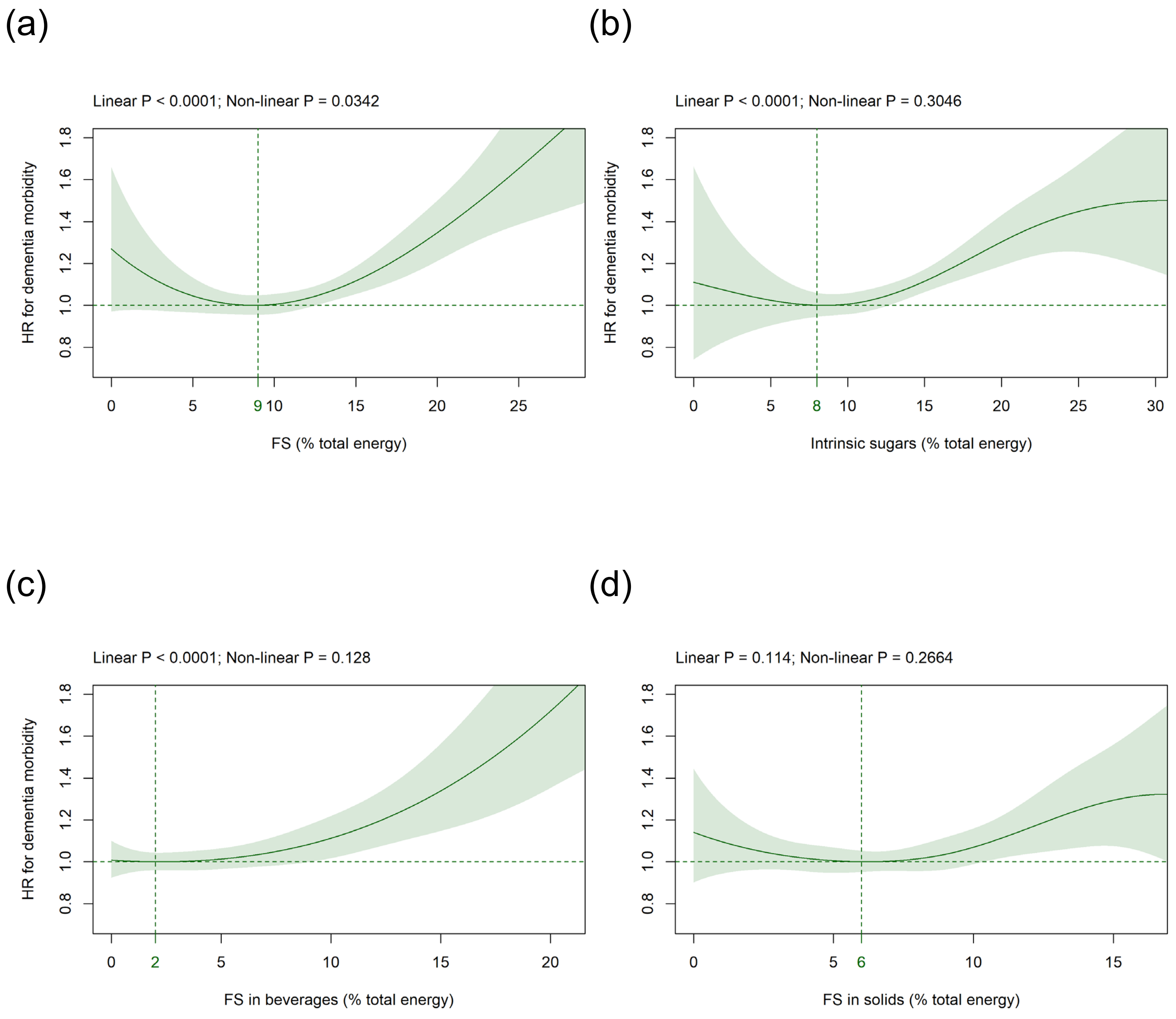

(a)
Linear P < 0.0001; Non-linear P = 0.0342
HR for dementia morbidity
0.8
1.0
1.2
1.4
1.6
1.8
0
5
9
10
15
20
25
FS (% total energy)
(b)
Linear P < 0.0001; Non-linear P = 0.3046
HR for dementia morbidity
0.8
1.0
1.2
1.4
1.6
1.8
0
5
8
10
15
20
25
30
Intrinsic sugars (% total energy)
(c)
Linear P < 0.0001; Non-linear P = 0.128
HR for dementia morbidity
0.8
1.0
1.2
1.4
1.6
1.8
0
2
5
10
15
20
FS in beverages (% total energy)
(d)
Linear P = 0.114; Non-linear P = 0.2664
0.8
1.0
1.2
1.4
1.6
1.8
0
5
6
10
15
FS in solids (% total energy)

Figure S4

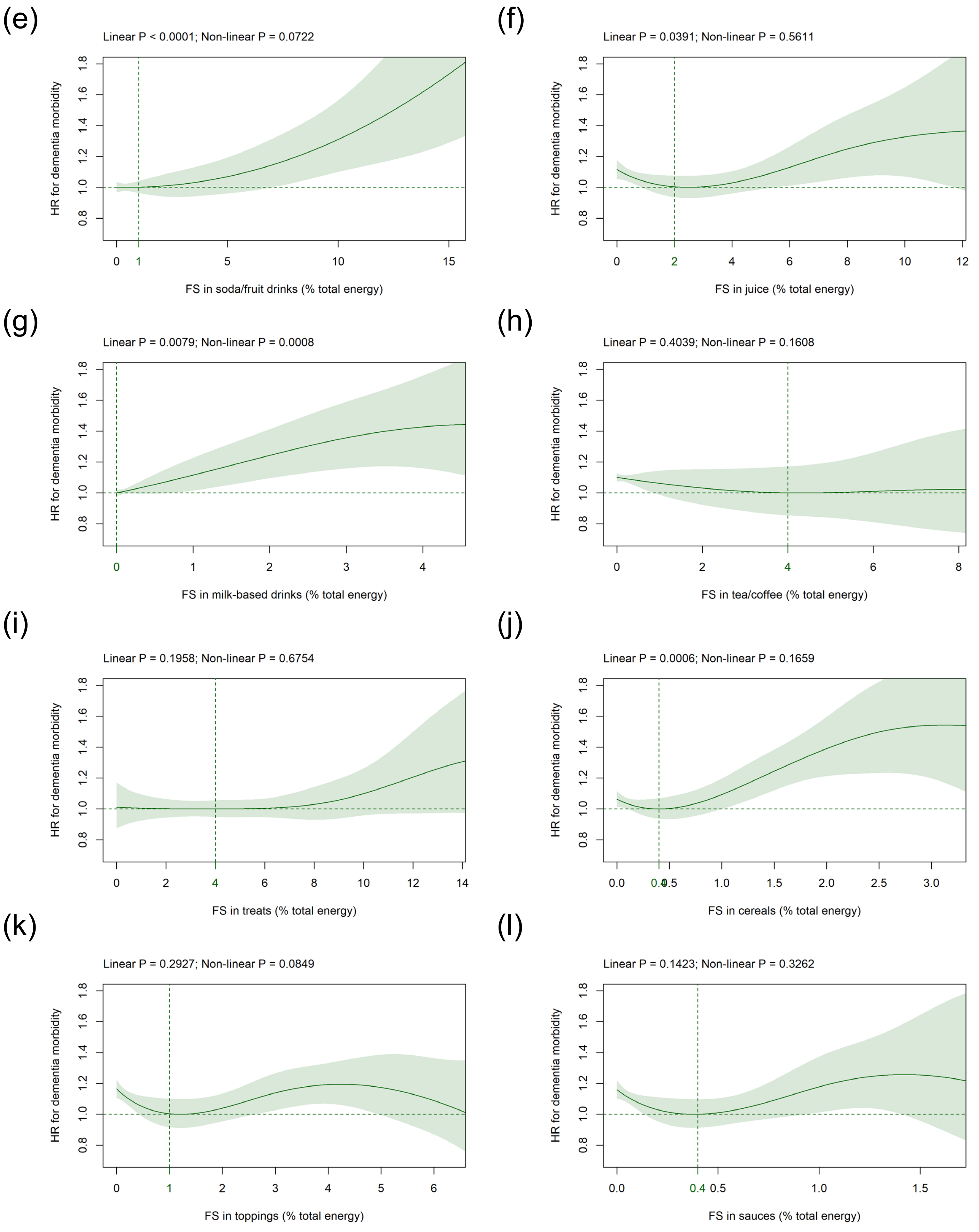

Figure S4 continued

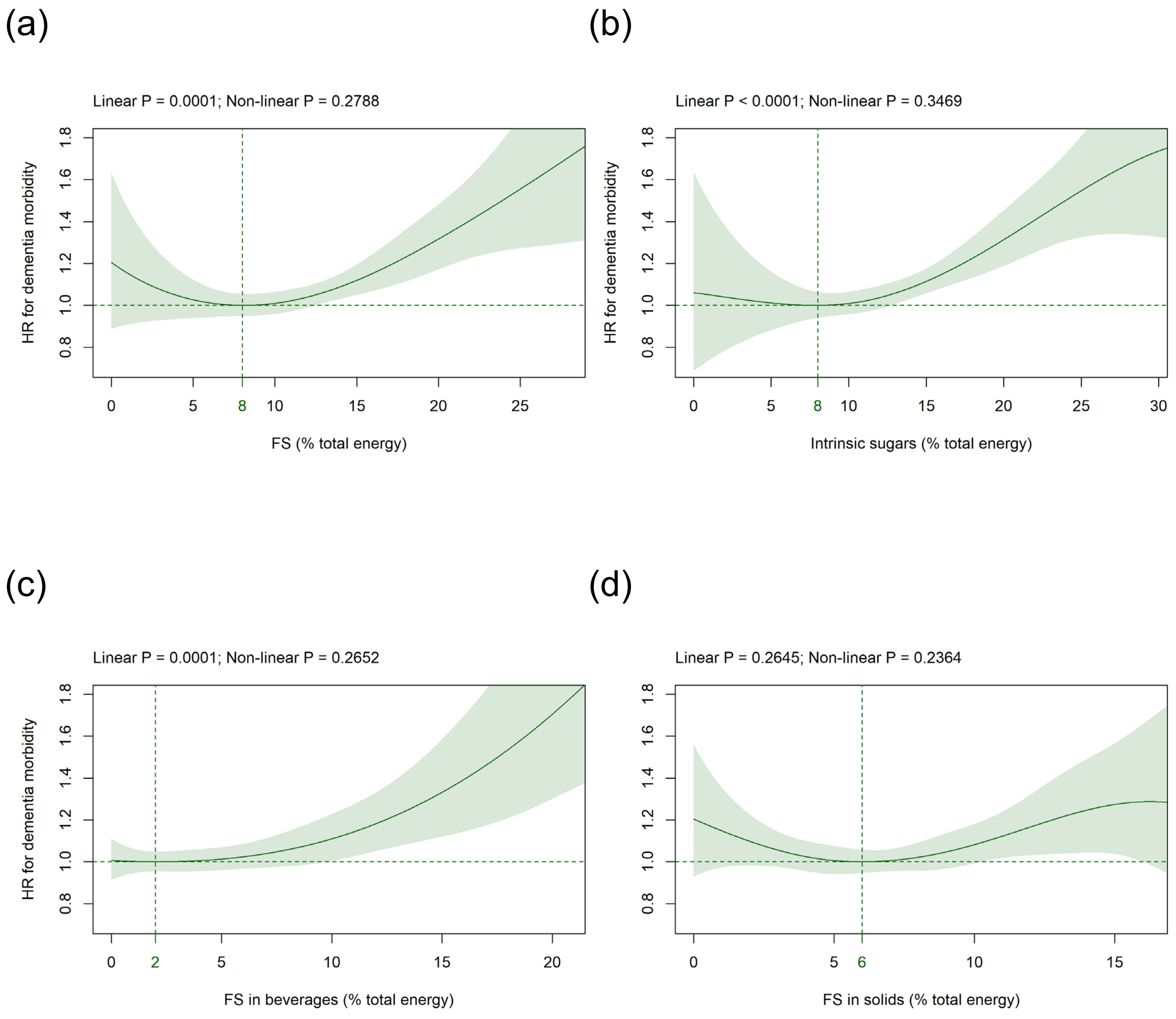
(a)
Linear P = 0.0001; Non-linear P = 0.2788
HR for dementia morbidity
0.8
1.0
1.2
1.4
1.6
1.8
0
5
8
10
15
20
25
FS (% total energy)
(b)
Linear P < 0.0001; Non-linear P = 0.3469
HR for dementia morbidity
0
5
8
10
15
20
25
30
Intrinsic sugars (% total energy)
(c)
Linear P = 0.0001; Non-linear P = 0.2652
HR for dementia morbidity
0
2
5
10
15
20
FS in beverages (% total energy)
(d)
Linear P = 0.2645; Non-linear P = 0.2364
0
5
6
10
15
FS in solids (% total energy)

Figure S5

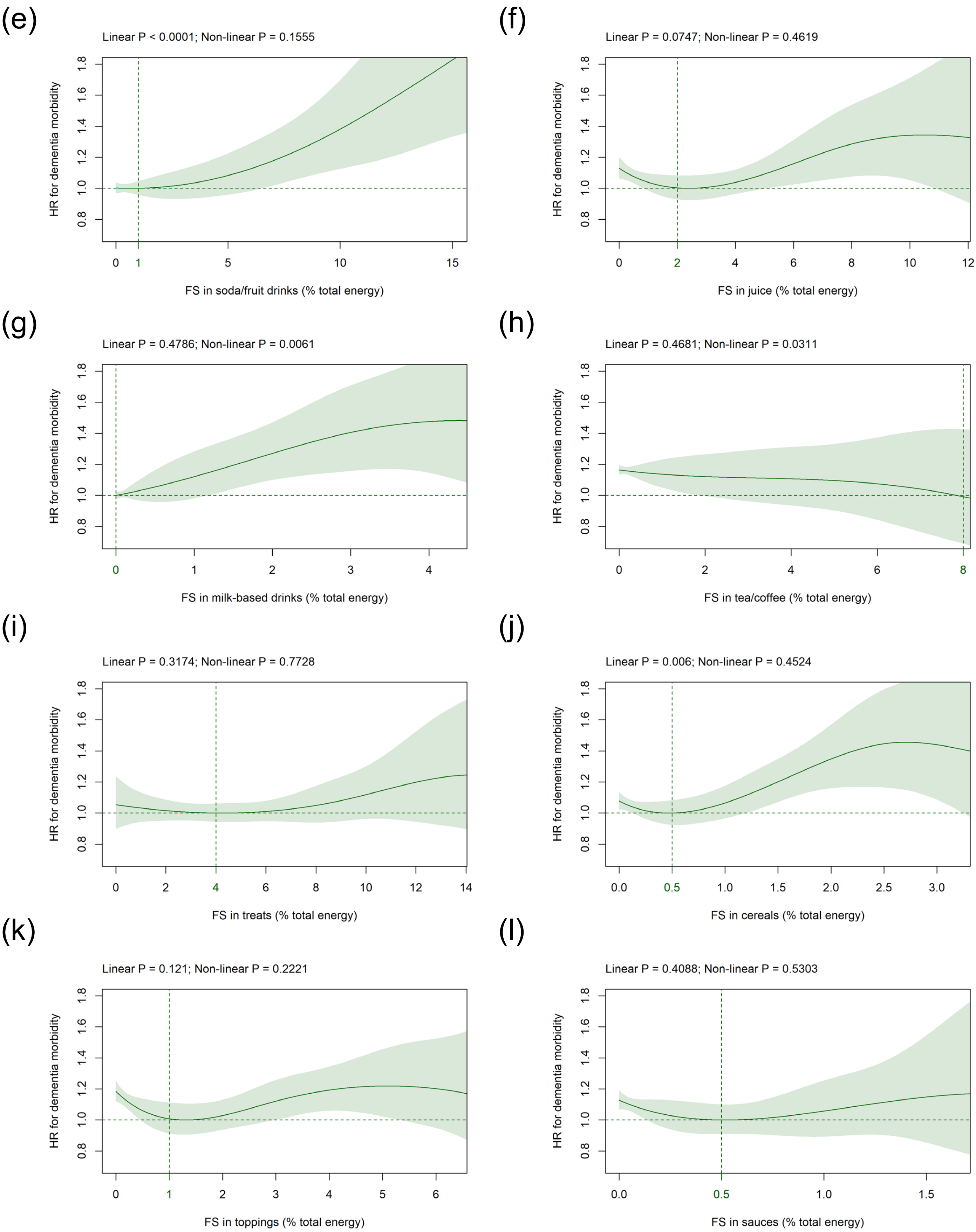

Figure S5 continued

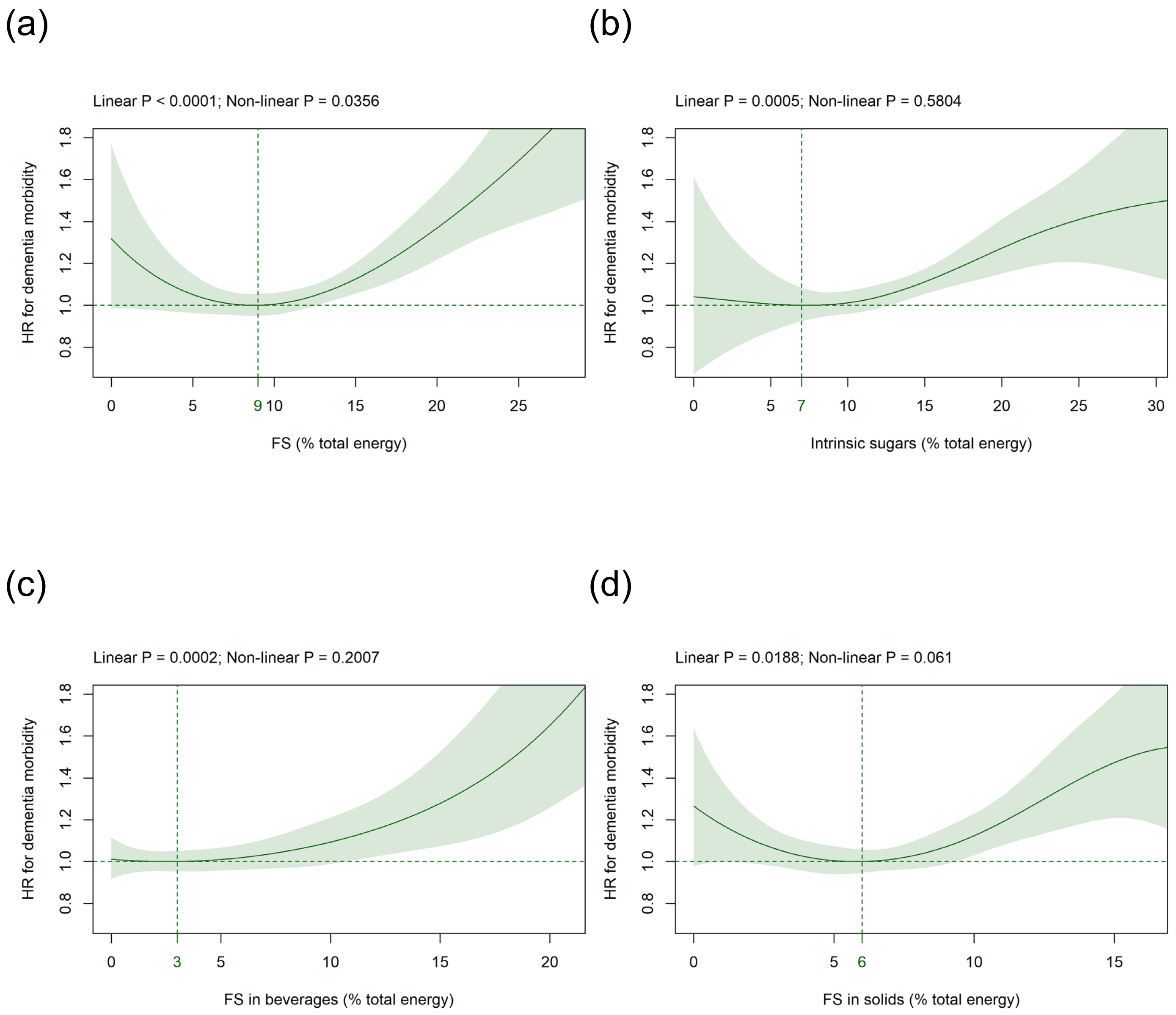

(a)
Linear P < 0.0001; Non-linear P = 0.0356
HR for dementia morbidity
0.8 1.0 1.2 1.4 1.6 1.8
0 5 9 10 15 20 25
FS (% total energy)
(b)
Linear P = 0.0005; Non-linear P = 0.5804
HR for dementia morbidity
0.8 1.0 1.2 1.4 1.6 1.8
0 5 7 10 15 20 25 30
Intrinsic sugars (% total energy)
(c)
Linear P = 0.0002; Non-linear P = 0.2007
HR for dementia morbidity
0.8 1.0 1.2 1.4 1.6 1.8
0 3 5 10 15 20
FS in beverages (% total energy)
(d)
Linear P = 0.0188; Non-linear P = 0.061
HR for dementia morbidity
0.8 1.0 1.2 1.4 1.6 1.8
0 5 6 10 15
FS in solids (% total energy)

Figure S6

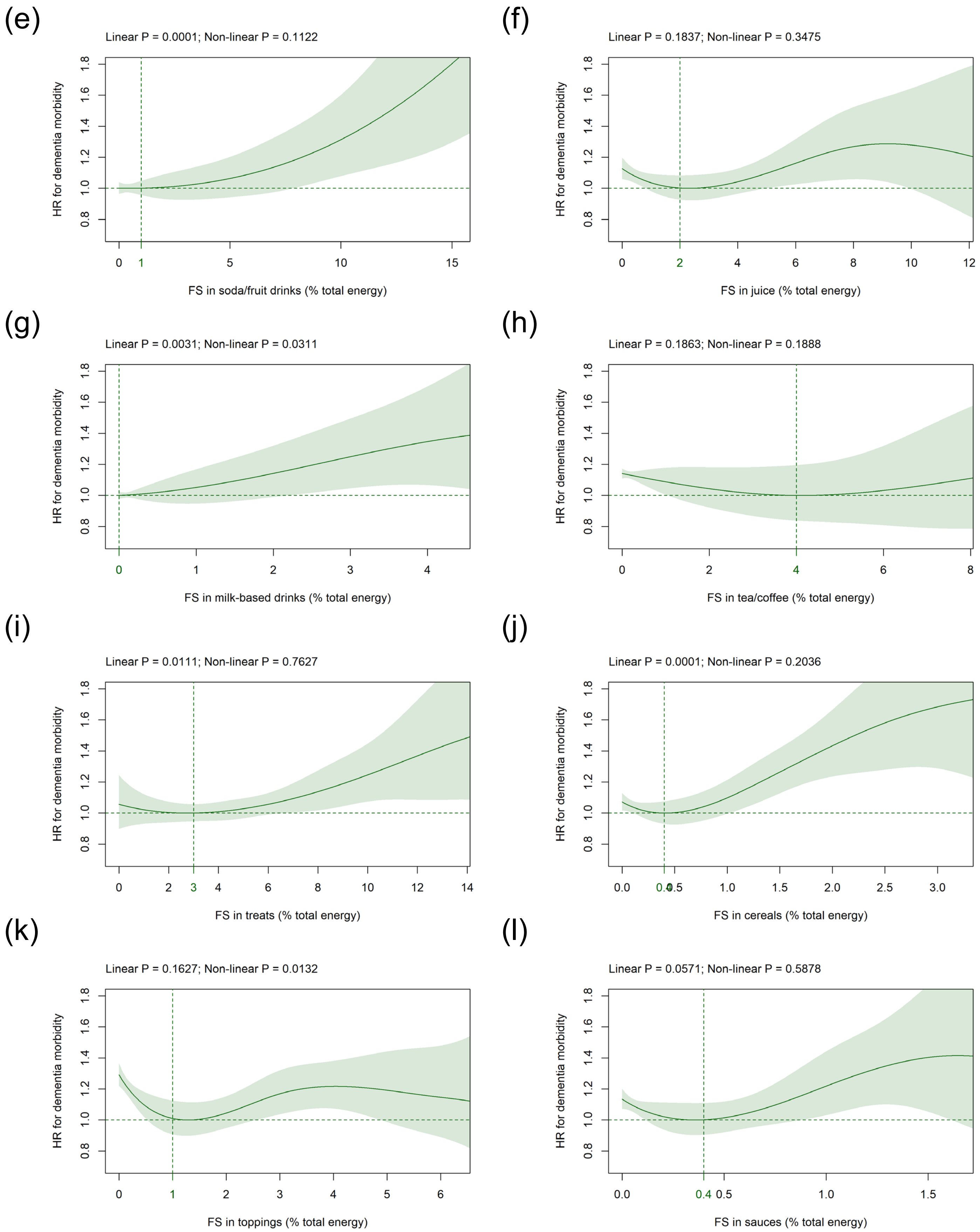

Figure S6 continued

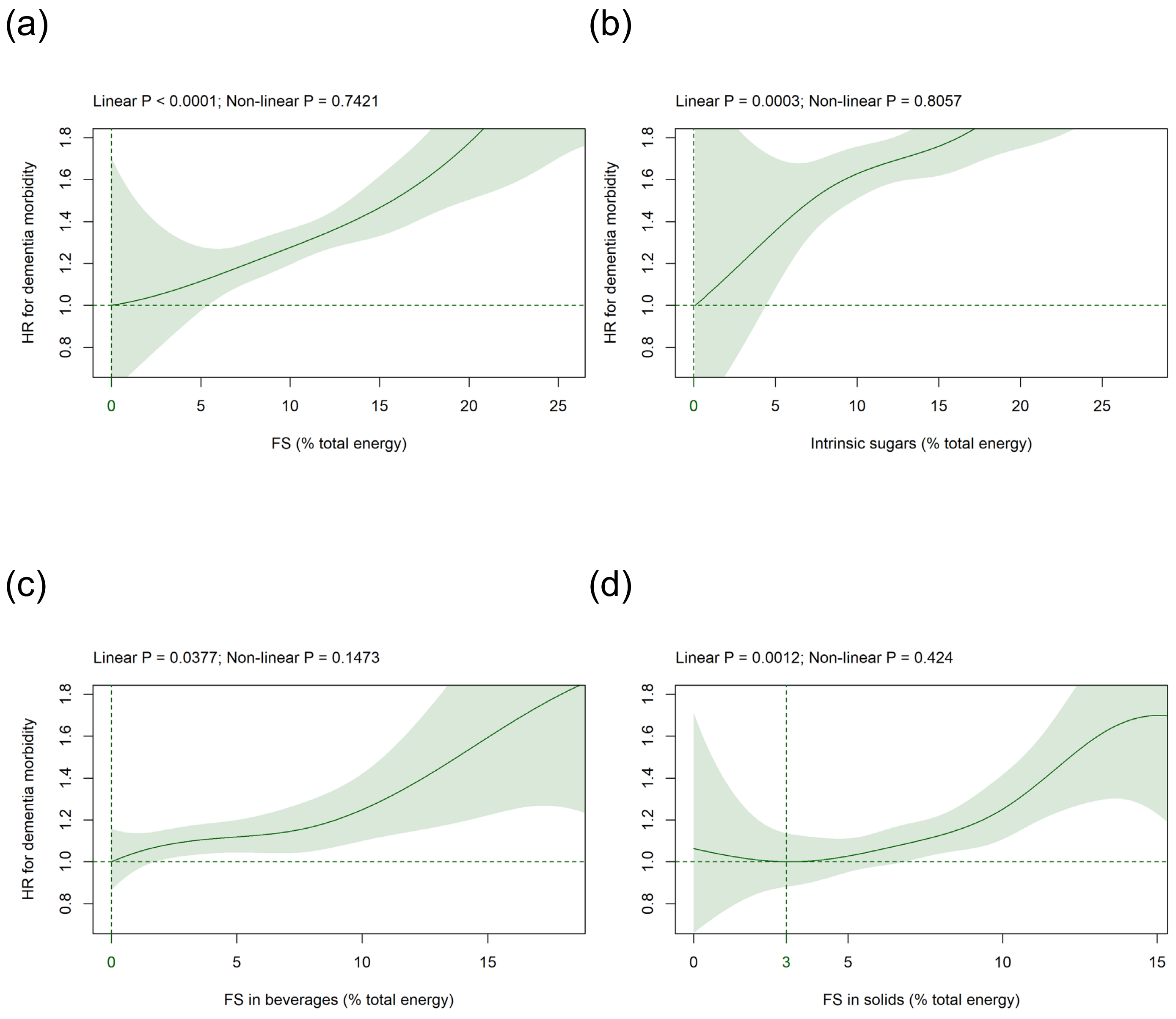
(a)
Linear P < 0.0001; Non-linear P = 0.7421
HR for dementia morbidity
0.8 1.0 1.2 1.4 1.6 1.8
0 5 10 15 20 25
FS (% total energy)
(b)
Linear P = 0.0003; Non-linear P = 0.8057
HR for dementia morbidity
0.8 1.0 1.2 1.4 1.6 1.8
0 5 10 15 20 25
Intrinsic sugars (% total energy)
(c)
Linear P = 0.0377; Non-linear P = 0.1473
HR for dementia morbidity
0.8 1.0 1.2 1.4 1.6 1.8
0 5 10 15
FS in beverages (% total energy)
(d)
Linear P = 0.0012; Non-linear P = 0.424
0.8 1.0 1.2 1.4 1.6 1.8
0 3 5 10 15
FS in solids (% total energy)

Figure S7

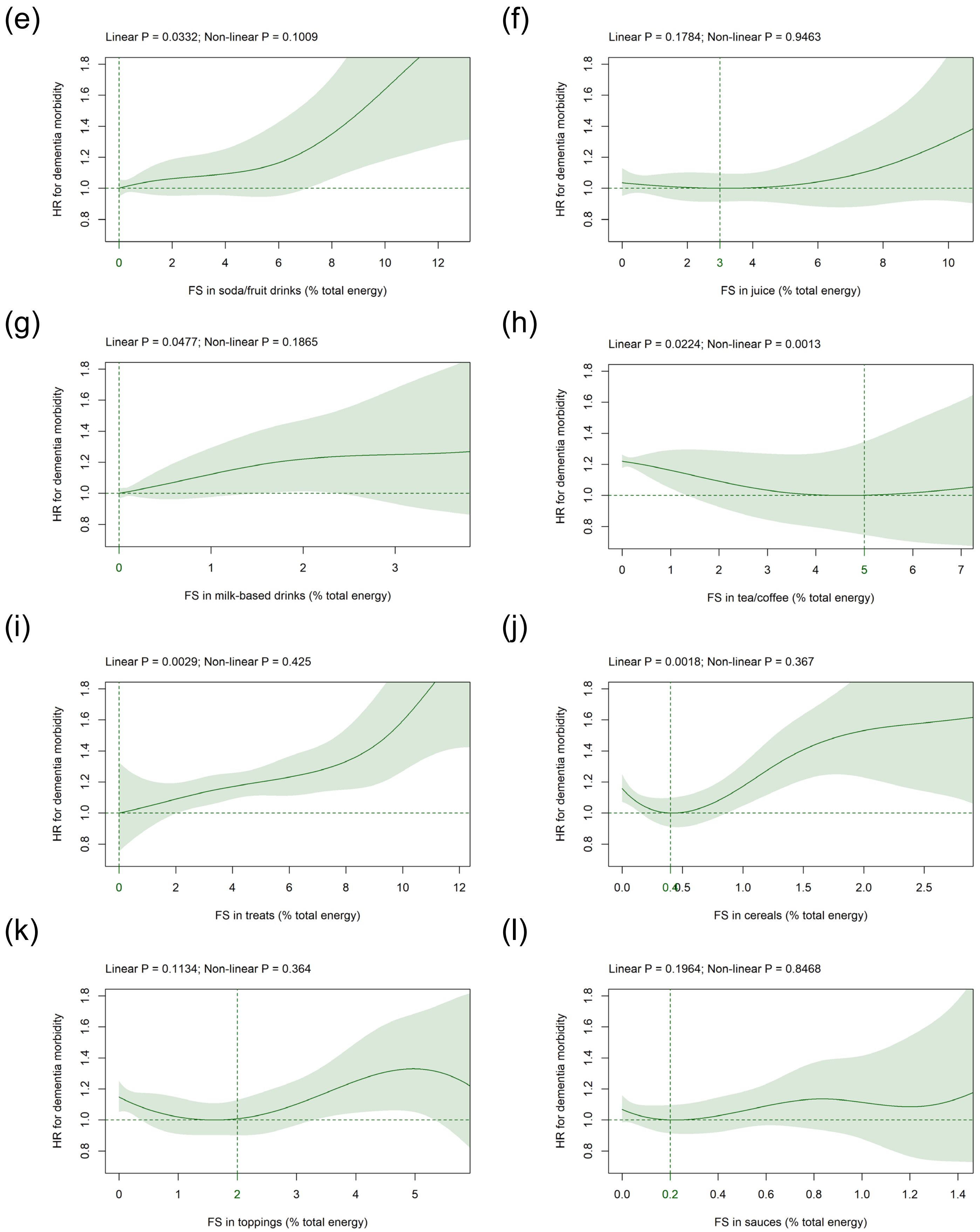

Figure S7 continued

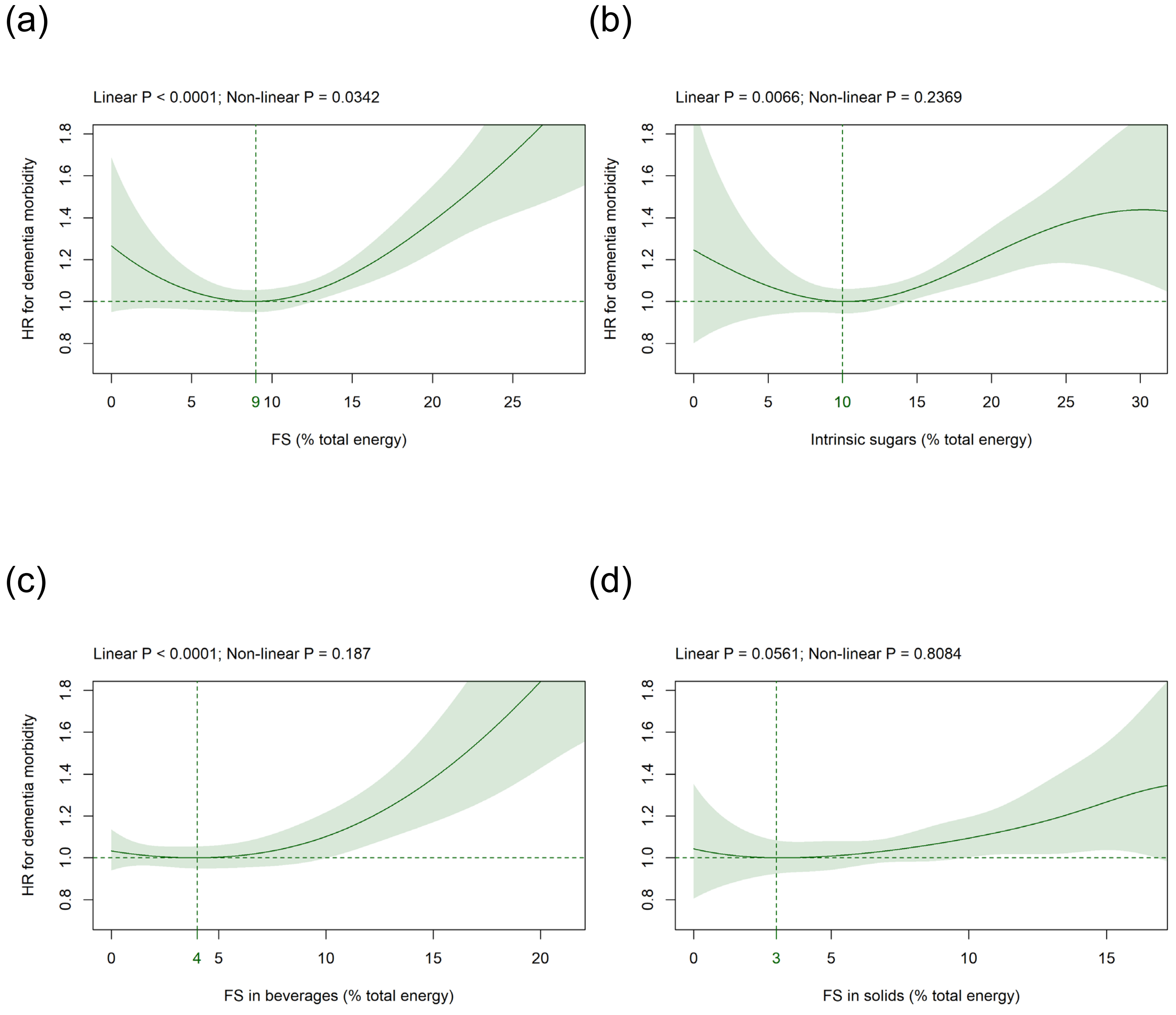
(a)
Linear P < 0.0001; Non-linear P = 0.0342
HR for dementia morbidity
0.8
1.0
1.2
1.4
1.6
1.8
0
5
9
10
15
20
25
FS (% total energy)
(b)
Linear P = 0.0066; Non-linear P = 0.2369
HR for dementia morbidity
0.8
1.0
1.2
1.4
1.6
1.8
0
5
10
15
20
25
30
Intrinsic sugars (% total energy)
(c)
Linear P < 0.0001; Non-linear P = 0.187
HR for dementia morbidity
0.8
1.0
1.2
1.4
1.6
1.8
0
4
5
10
15
20
FS in beverages (% total energy)
(d)
Linear P = 0.0561; Non-linear P = 0.8084
0.8
1.0
1.2
1.4
1.6
1.8
0
3
5
10
15
FS in solids (% total energy)

Figure S8

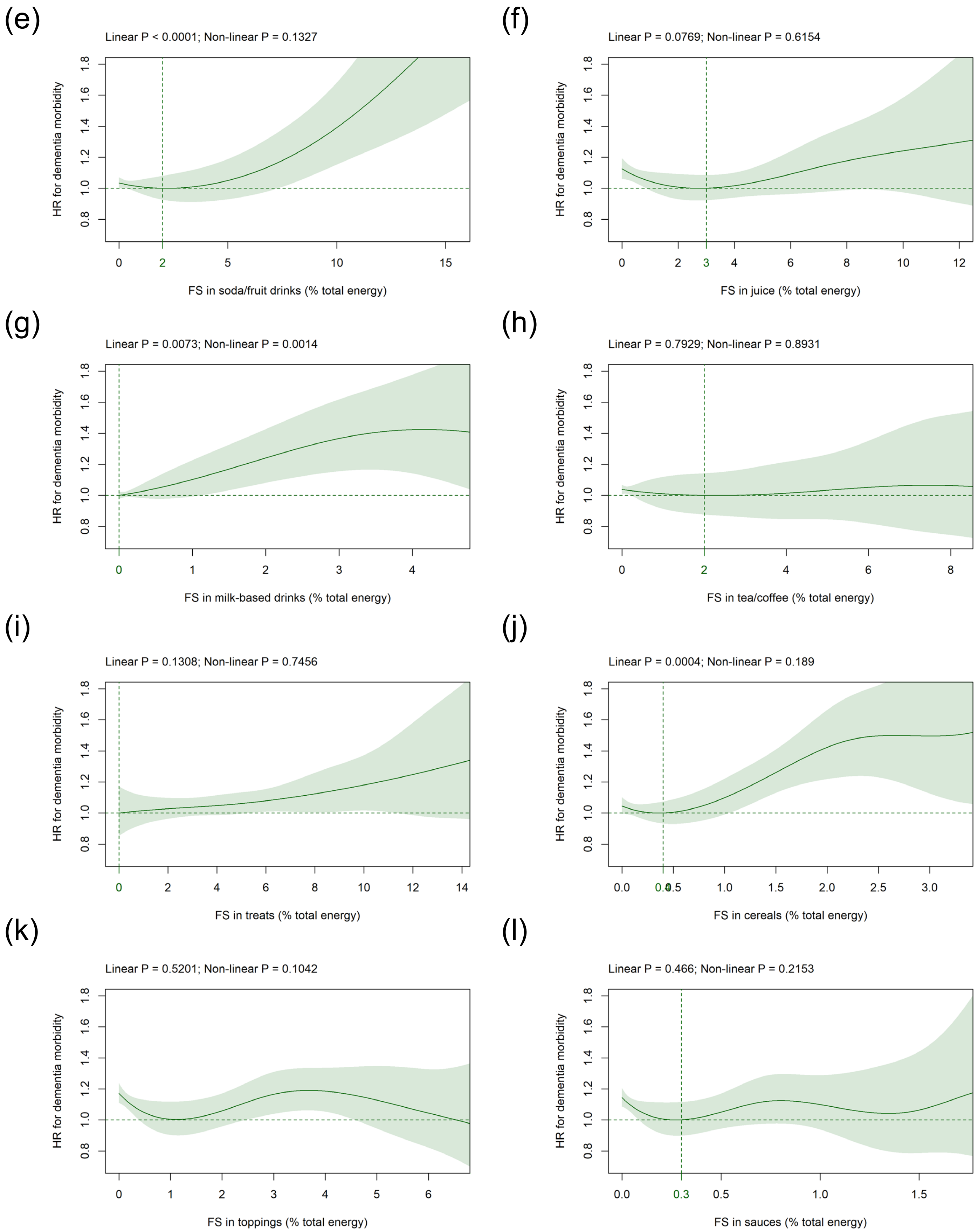

Figure S8 continued

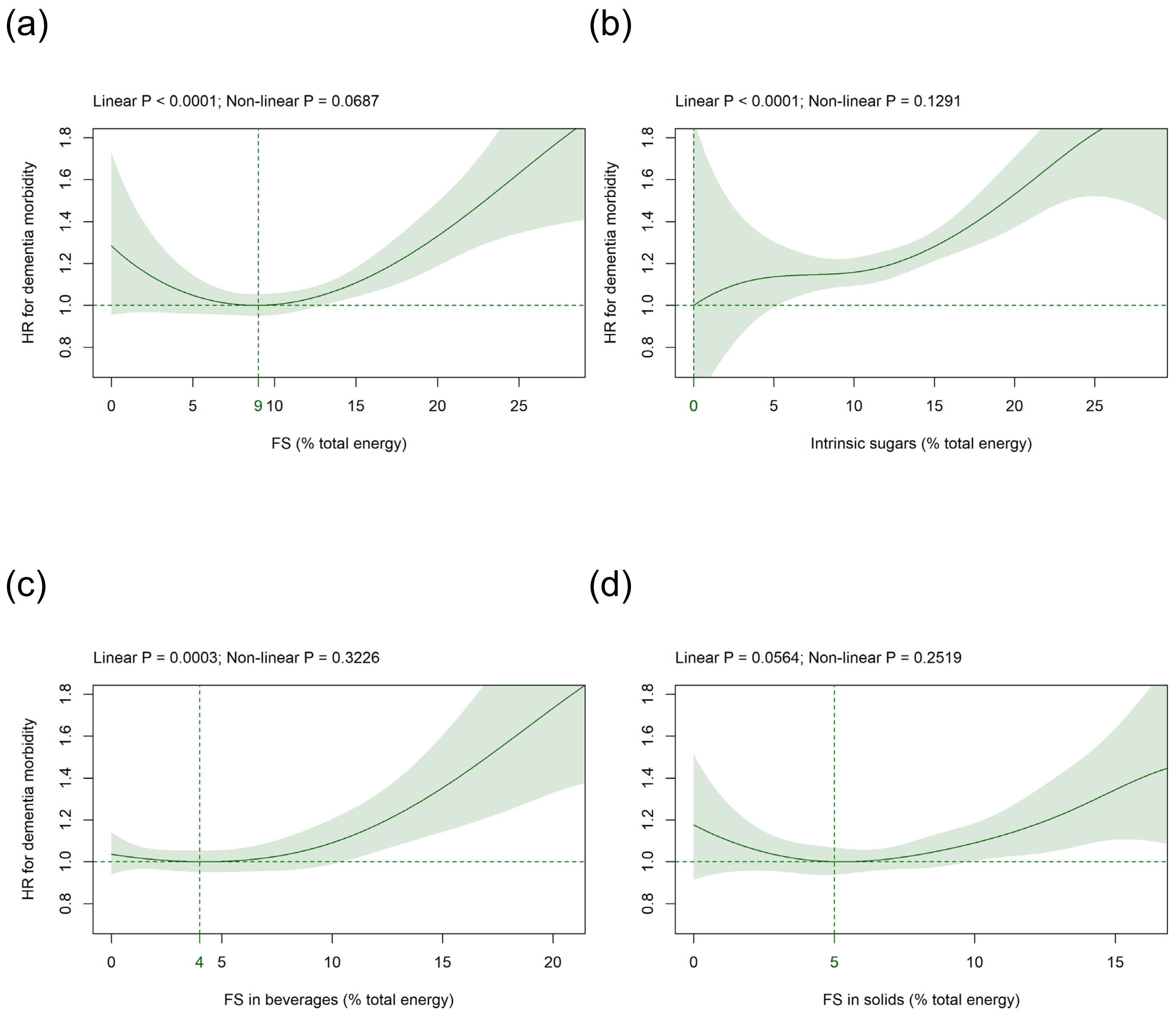
(a)
Linear P < 0.0001; Non-linear P = 0.0687
HR for dementia morbidity
FS (% total energy)
(b)
Linear P < 0.0001; Non-linear P = 0.1291
HR for dementia morbidity
Intrinsic sugars (% total energy)
(c)
Linear P = 0.0003; Non-linear P = 0.3226
HR for dementia morbidity
FS in beverages (% total energy)
(d)
Linear P = 0.0564; Non-linear P = 0.2519
FS in solids (% total energy)

Figure S9

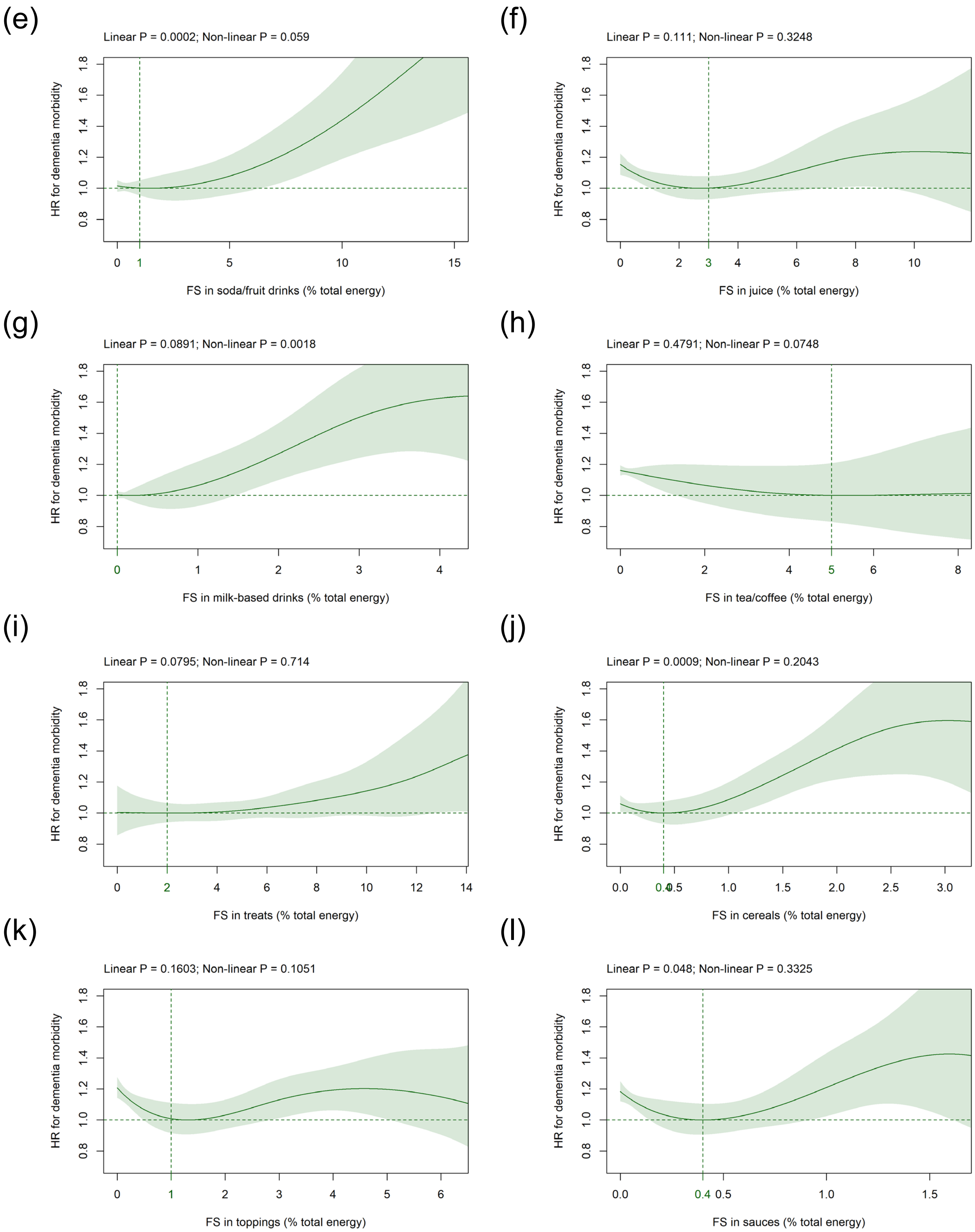

Figure S9 continued

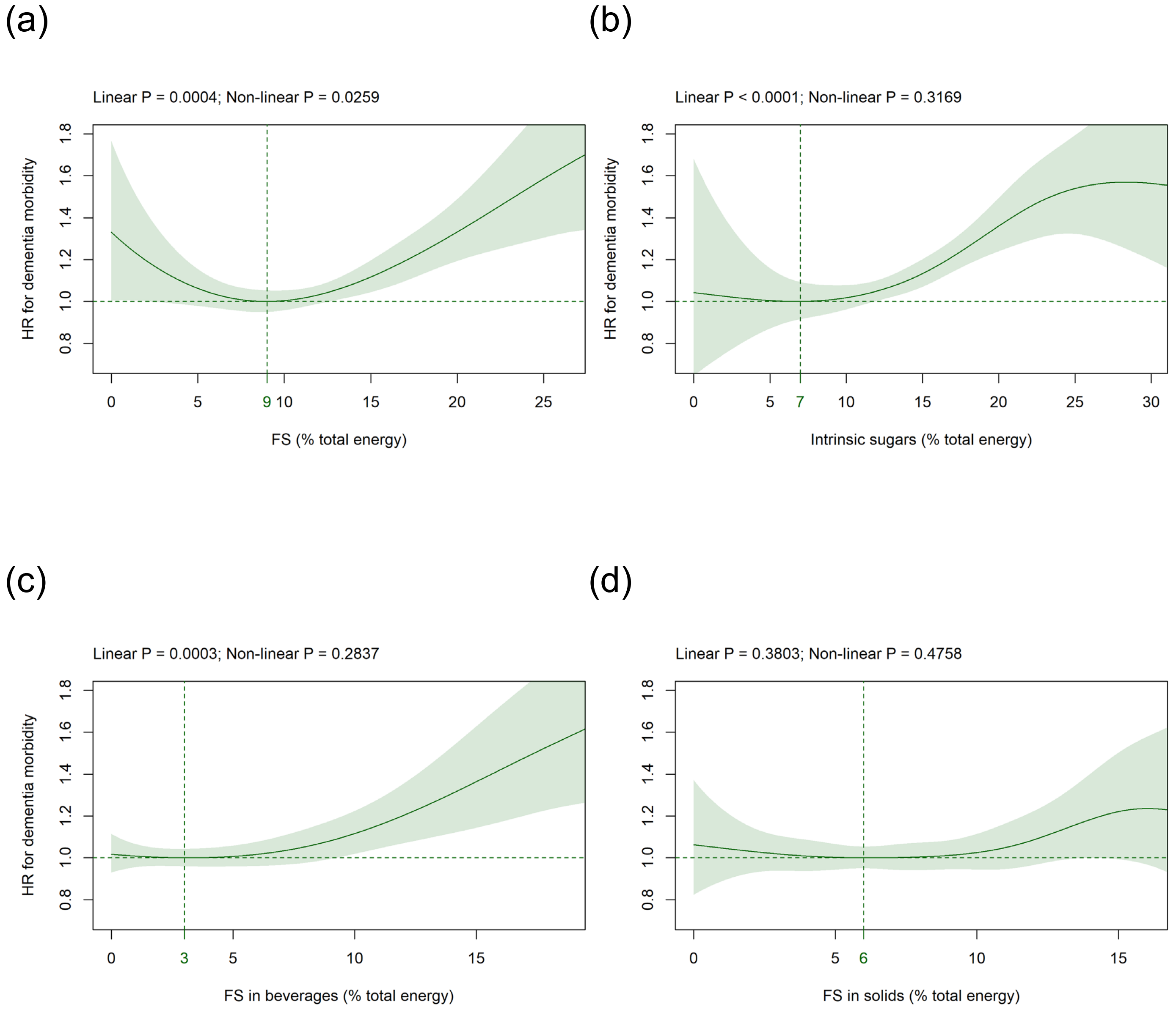
(a)
Linear P = 0.0004; Non-linear P = 0.0259
HR for dementia morbidity
0.8
1.0
1.2
1.4
1.6
1.8
0
5
9
10
15
20
25
FS (% total energy)
(b)
Linear P < 0.0001; Non-linear P = 0.3169
HR for dementia morbidity
0.8
1.0
1.2
1.4
1.6
1.8
0
5
7
10
15
20
25
30
Intrinsic sugars (% total energy)
(c)
Linear P = 0.0003; Non-linear P = 0.2837
HR for dementia morbidity
0.8
1.0
1.2
1.4
1.6
1.8
0
3
5
10
15
FS in beverages (% total energy)
(d)
Linear P = 0.3803; Non-linear P = 0.4758
0.8
1.0
1.2
1.4
1.6
1.8
0
5
6
10
15
FS in solids (% total energy)

Figure S10

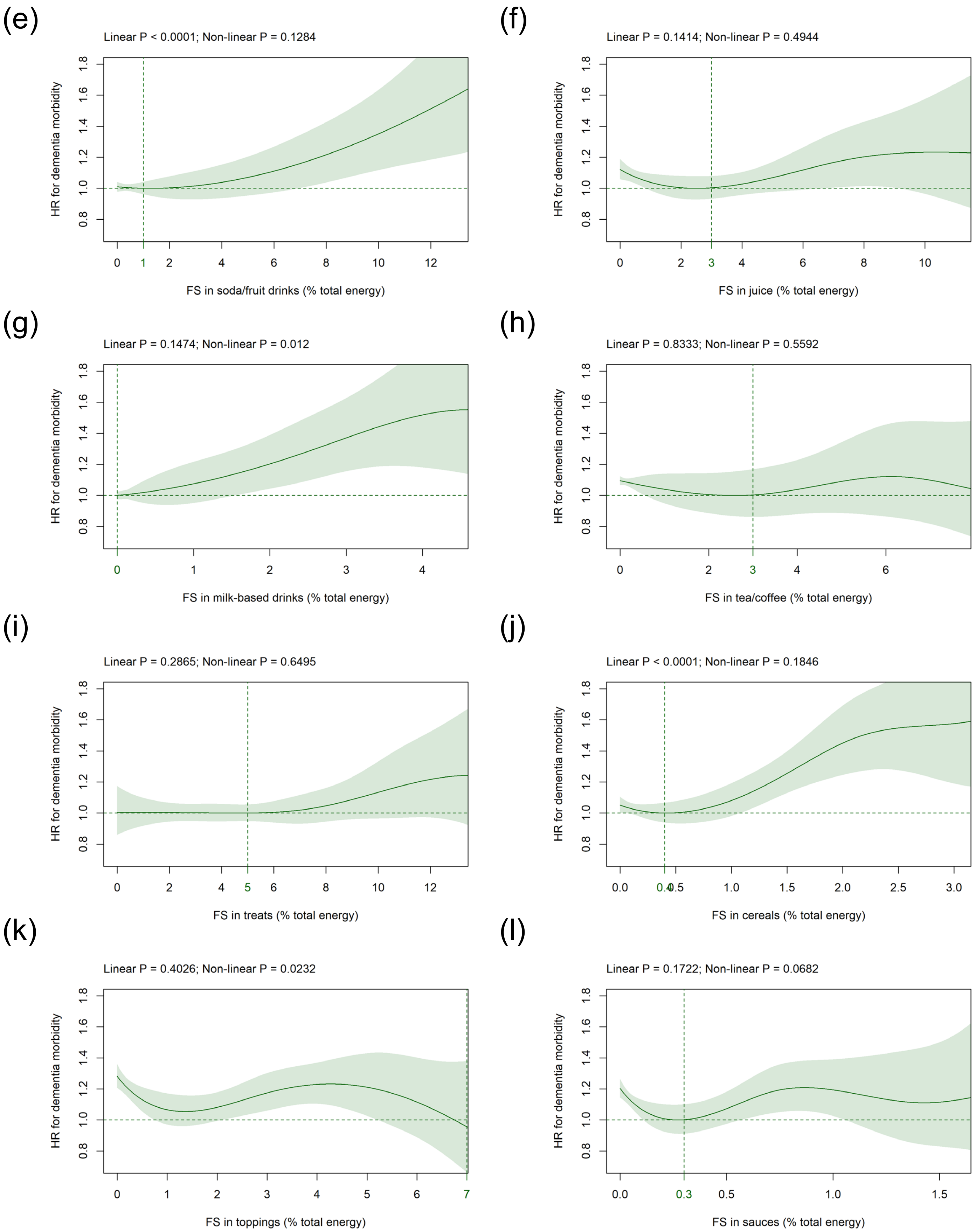

Figure S10 continued

(a)

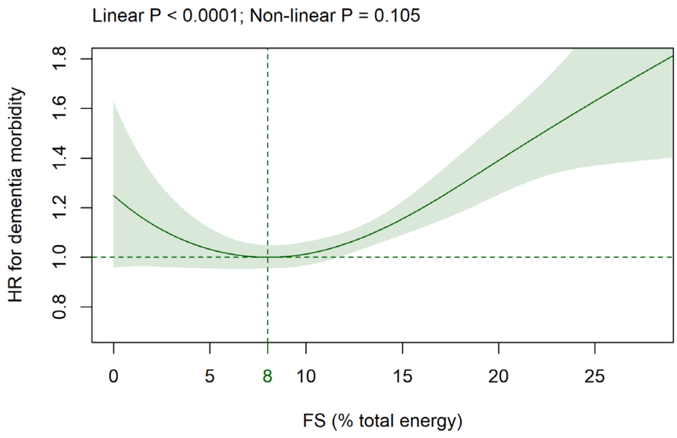

(b)

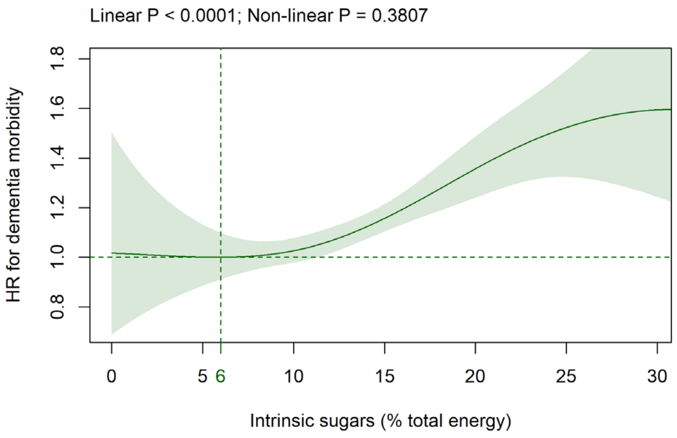

(c)

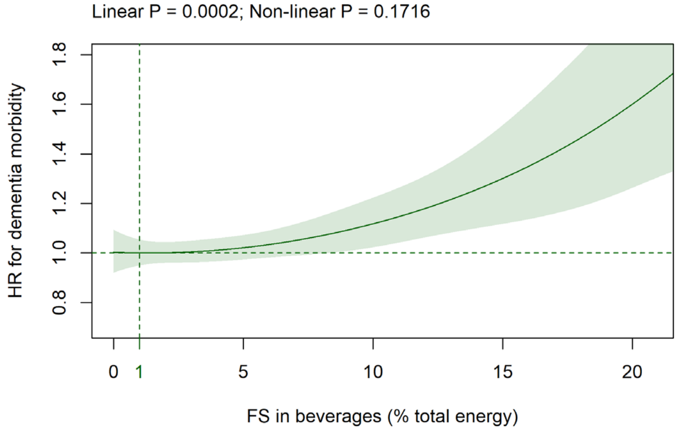

(d)

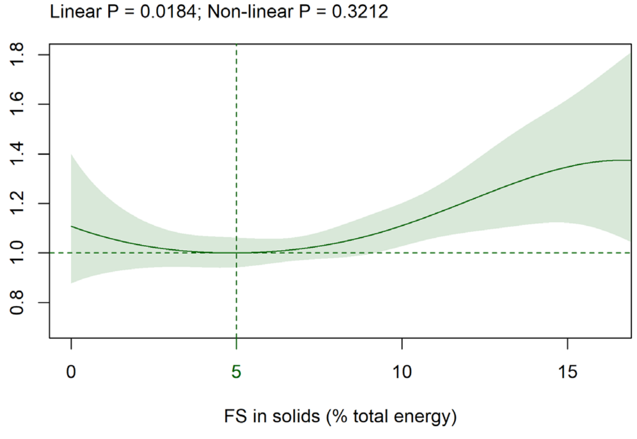

Figure S11

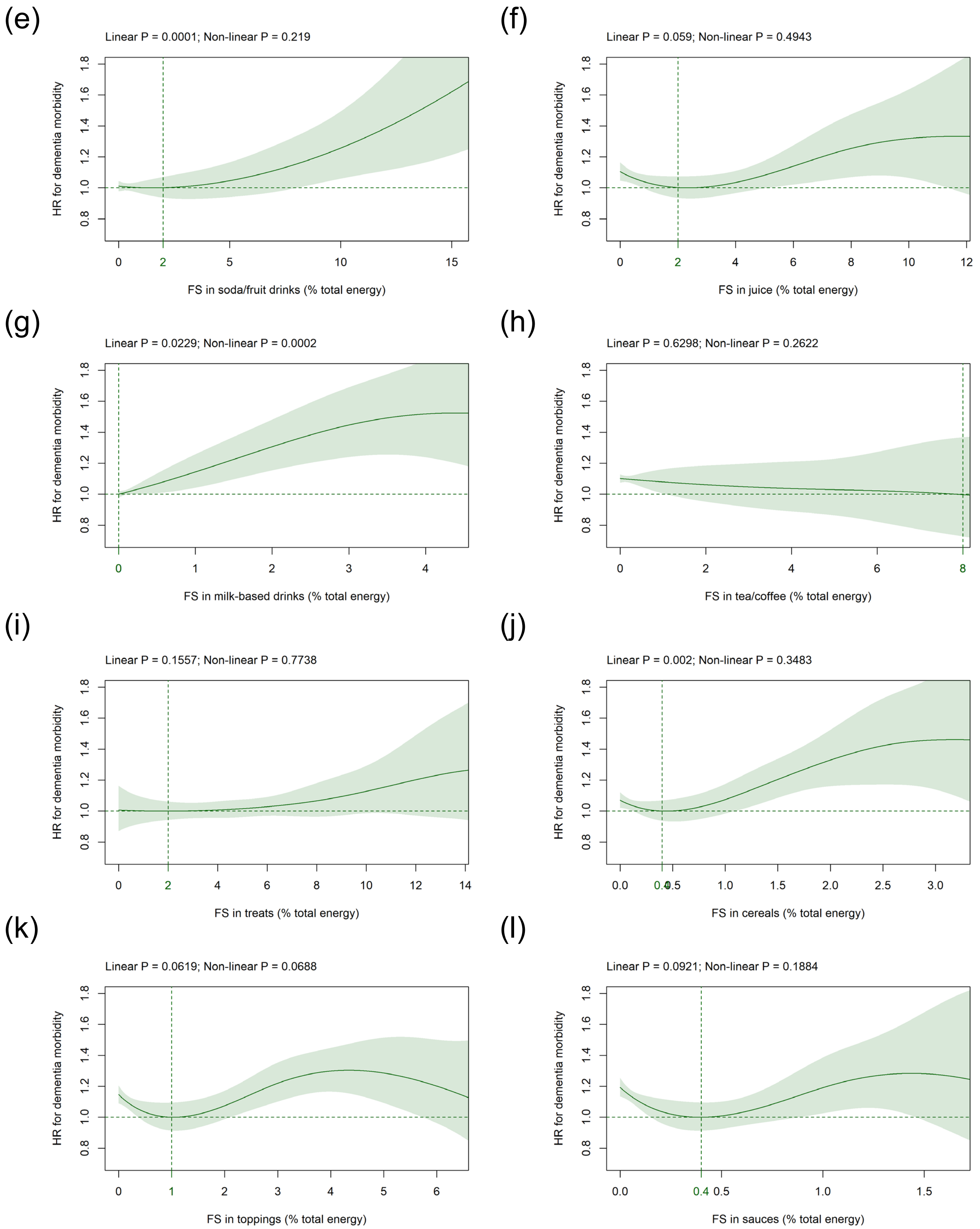

Figure S11 continued

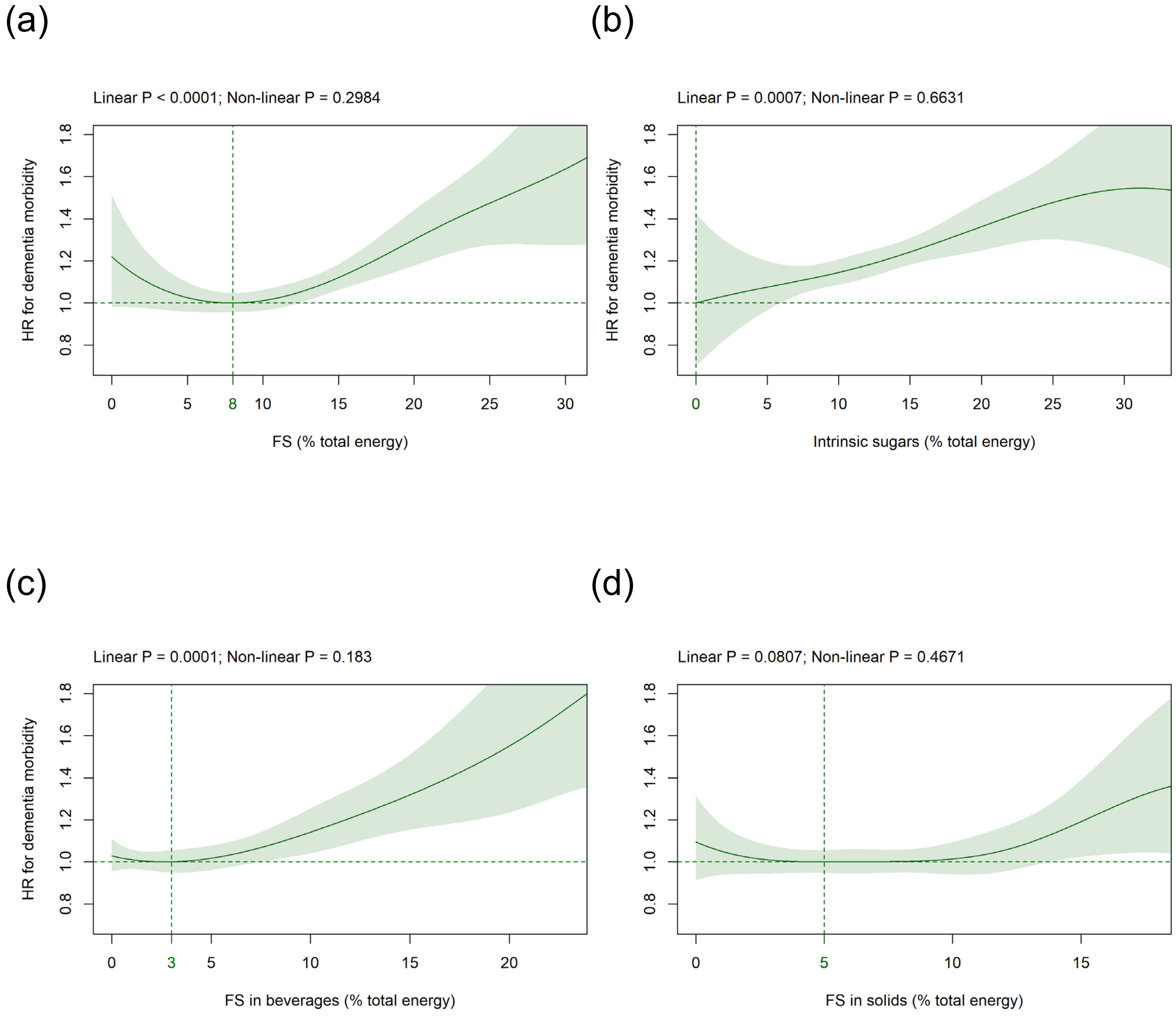
(a)
Linear P < 0.0001; Non-linear P = 0.2984
HR for dementia morbidity
0.8 1.0 1.2 1.4 1.6 1.8
0 5 8 10 15 20 25 30
FS (% total energy)
(b)
Linear P = 0.0007; Non-linear P = 0.6631
HR for dementia morbidity
0.8 1.0 1.2 1.4 1.6 1.8
0 5 10 15 20 25 30
Intrinsic sugars (% total energy)
(c)
Linear P = 0.0001; Non-linear P = 0.183
HR for dementia morbidity
0.8 1.0 1.2 1.4 1.6 1.8
0 3 5 10 15 20
FS in beverages (% total energy)
(d)
Linear P = 0.0807; Non-linear P = 0.4671
0.8 1.0 1.2 1.4 1.6 1.8
0 5 10 15
FS in solids (% total energy)

Figure S12

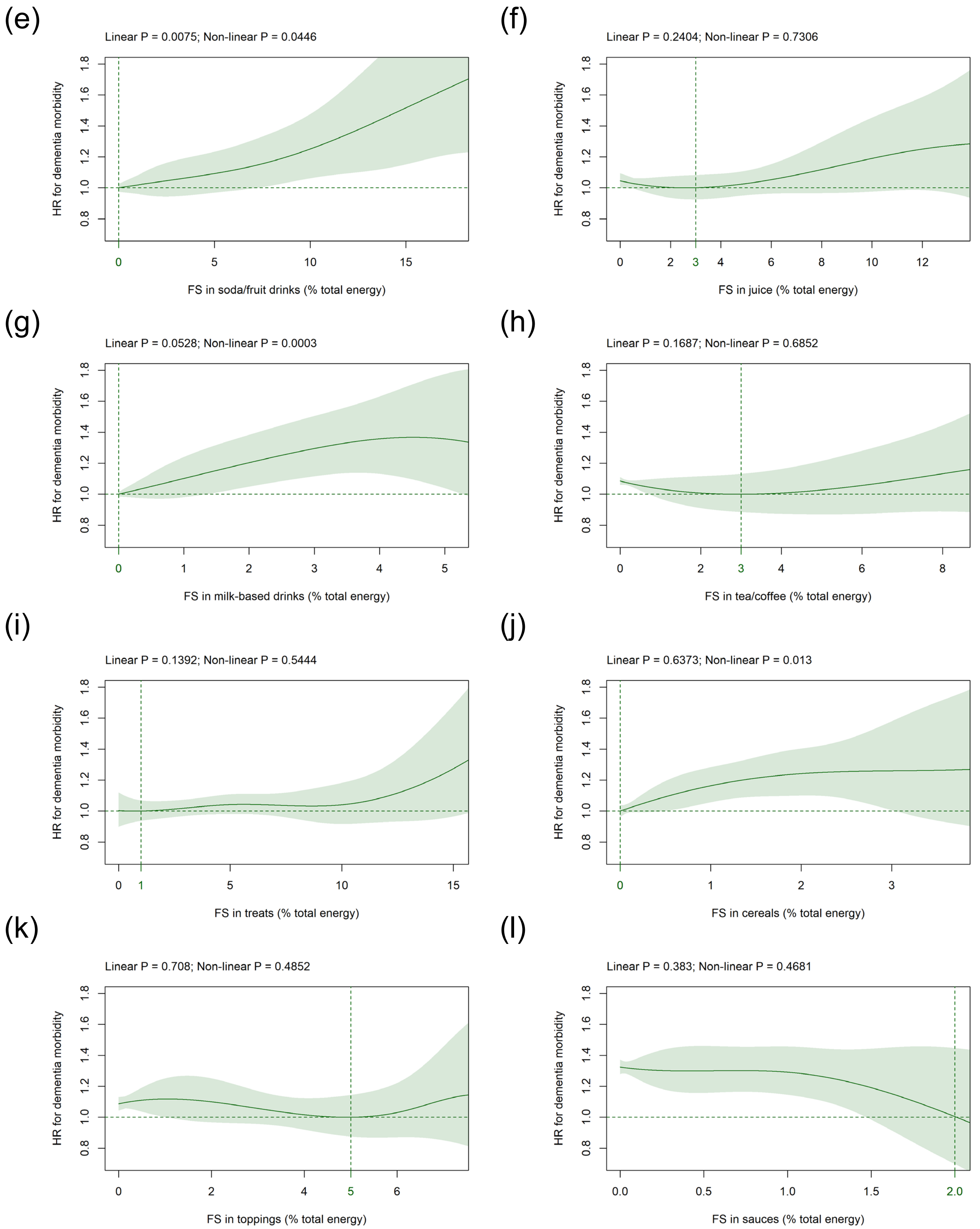

Figure S12 continued

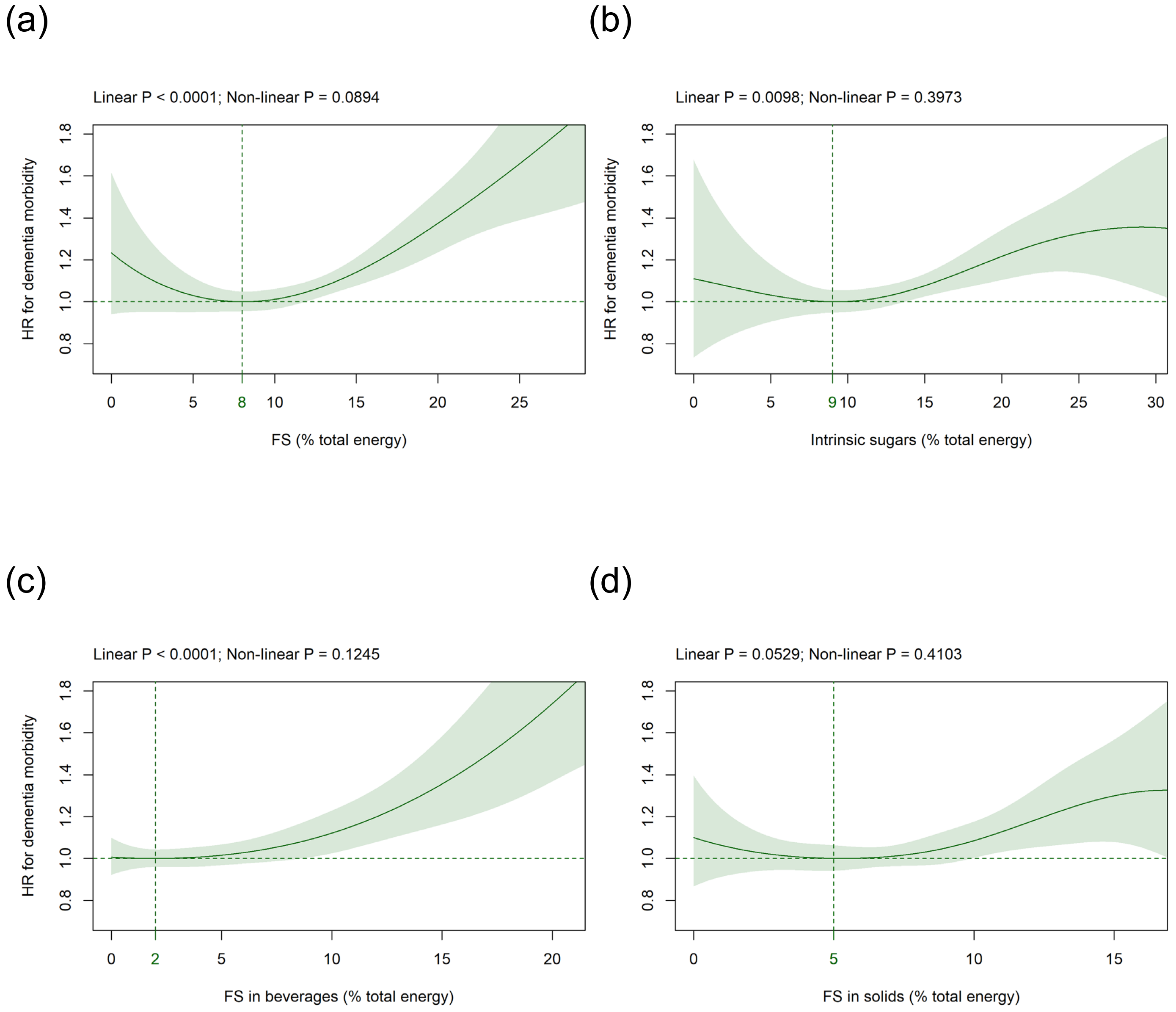
(a)
Linear P < 0.0001; Non-linear P = 0.0894
HR for dementia morbidity
FS (% total energy)
(b)
Linear P = 0.0098; Non-linear P = 0.3973
HR for dementia morbidity
Intrinsic sugars (% total energy)
(c)
Linear P < 0.0001; Non-linear P = 0.1245
HR for dementia morbidity
FS in beverages (% total energy)
(d)
Linear P = 0.0529; Non-linear P = 0.4103
FS in solids (% total energy)

Figure S13

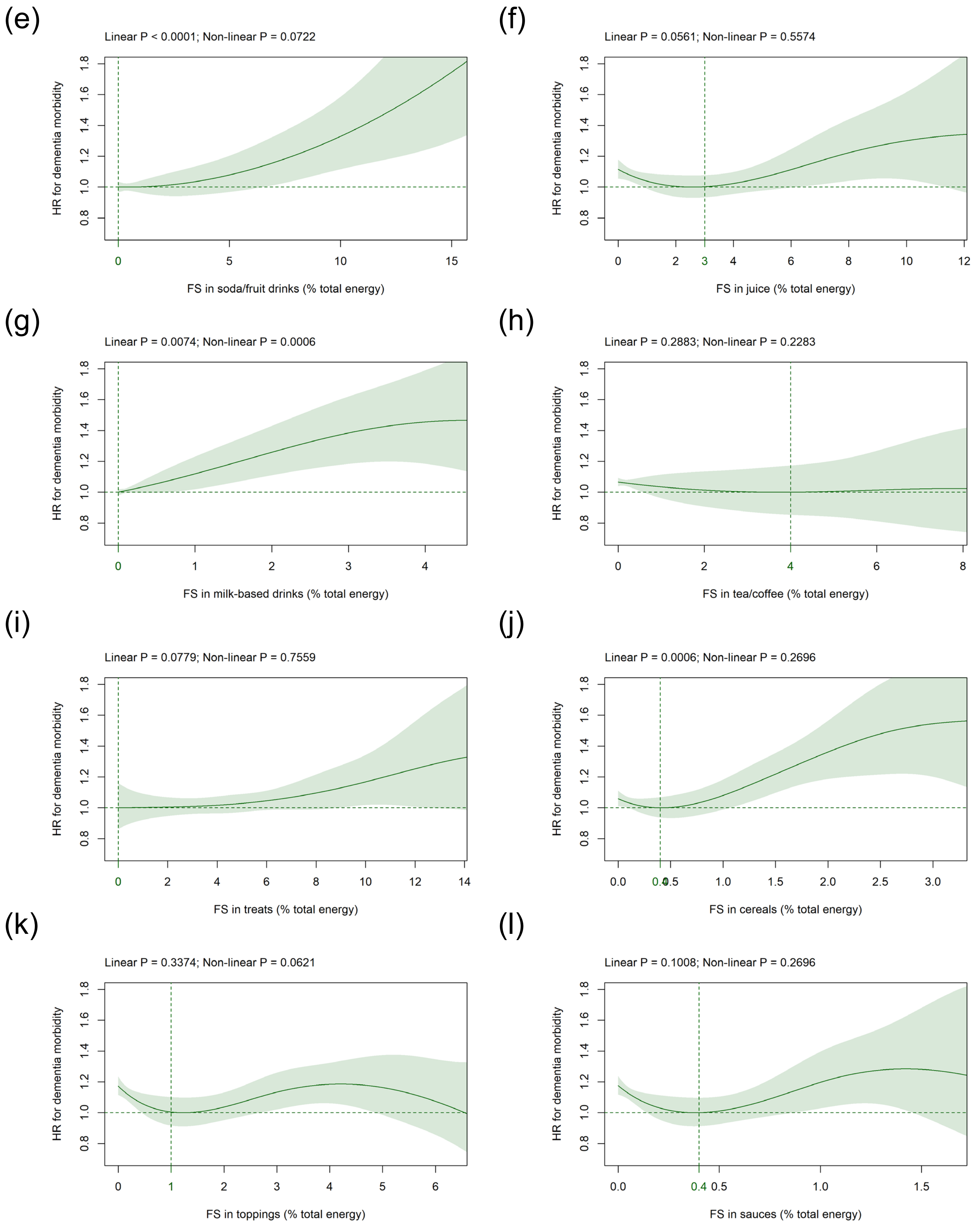

Figure S13 continued

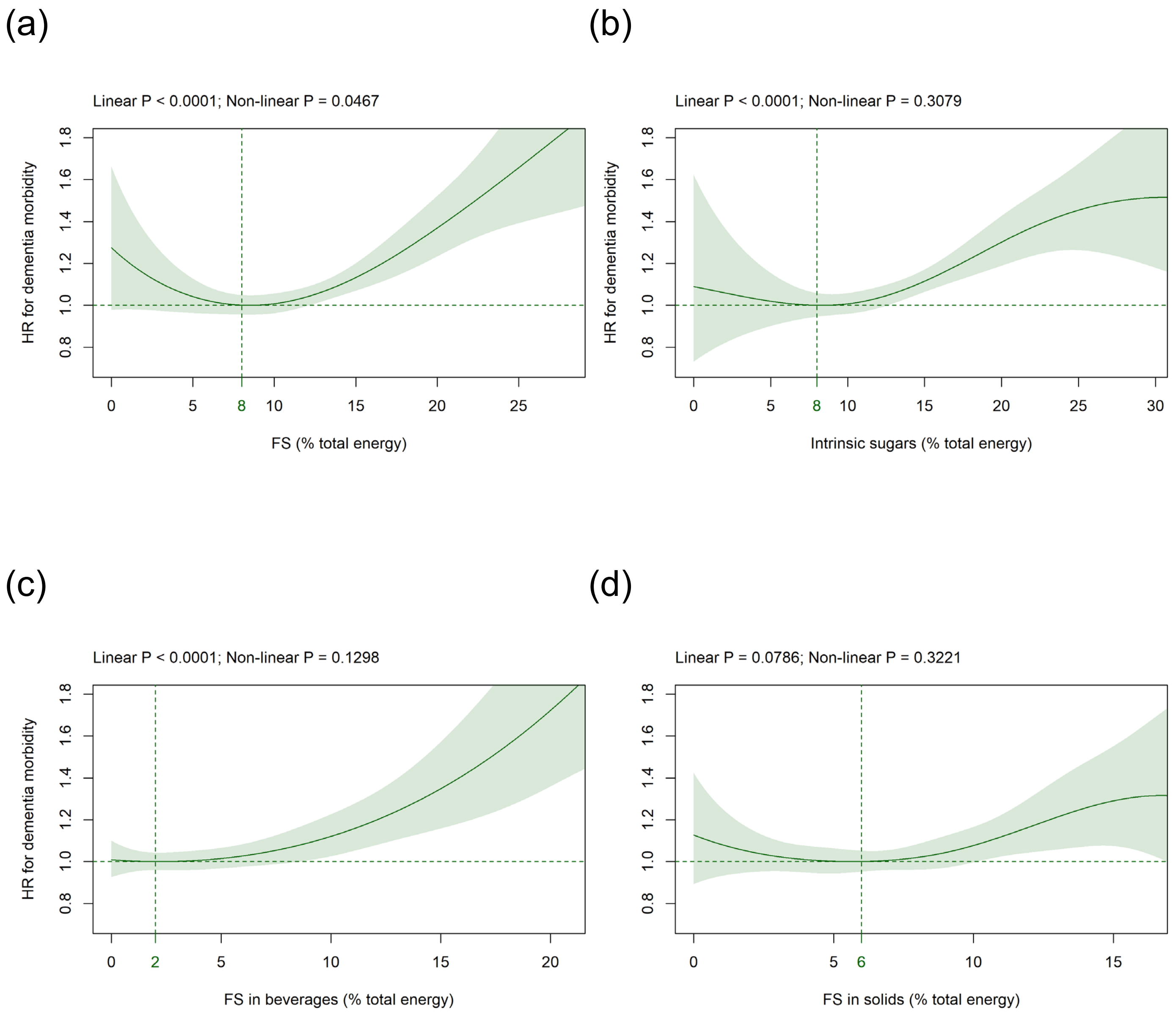
(a)
Linear P < 0.0001; Non-linear P = 0.0467
HR for dementia morbidity
0.8 1.0 1.2 1.4 1.6 1.8
0 5 8 10 15 20 25
FS (% total energy)
(b)
Linear P < 0.0001; Non-linear P = 0.3079
HR for dementia morbidity
0.8 1.0 1.2 1.4 1.6 1.8
0 5 8 10 15 20 25 30
Intrinsic sugars (% total energy)
(c)
Linear P < 0.0001; Non-linear P = 0.1298
HR for dementia morbidity
0.8 1.0 1.2 1.4 1.6 1.8
0 2 5 10 15 20
FS in beverages (% total energy)
(d)
Linear P = 0.0786; Non-linear P = 0.3221
0.8 1.0 1.2 1.4 1.6 1.8
0 5 6 10 15
FS in solids (% total energy)

Figure S14

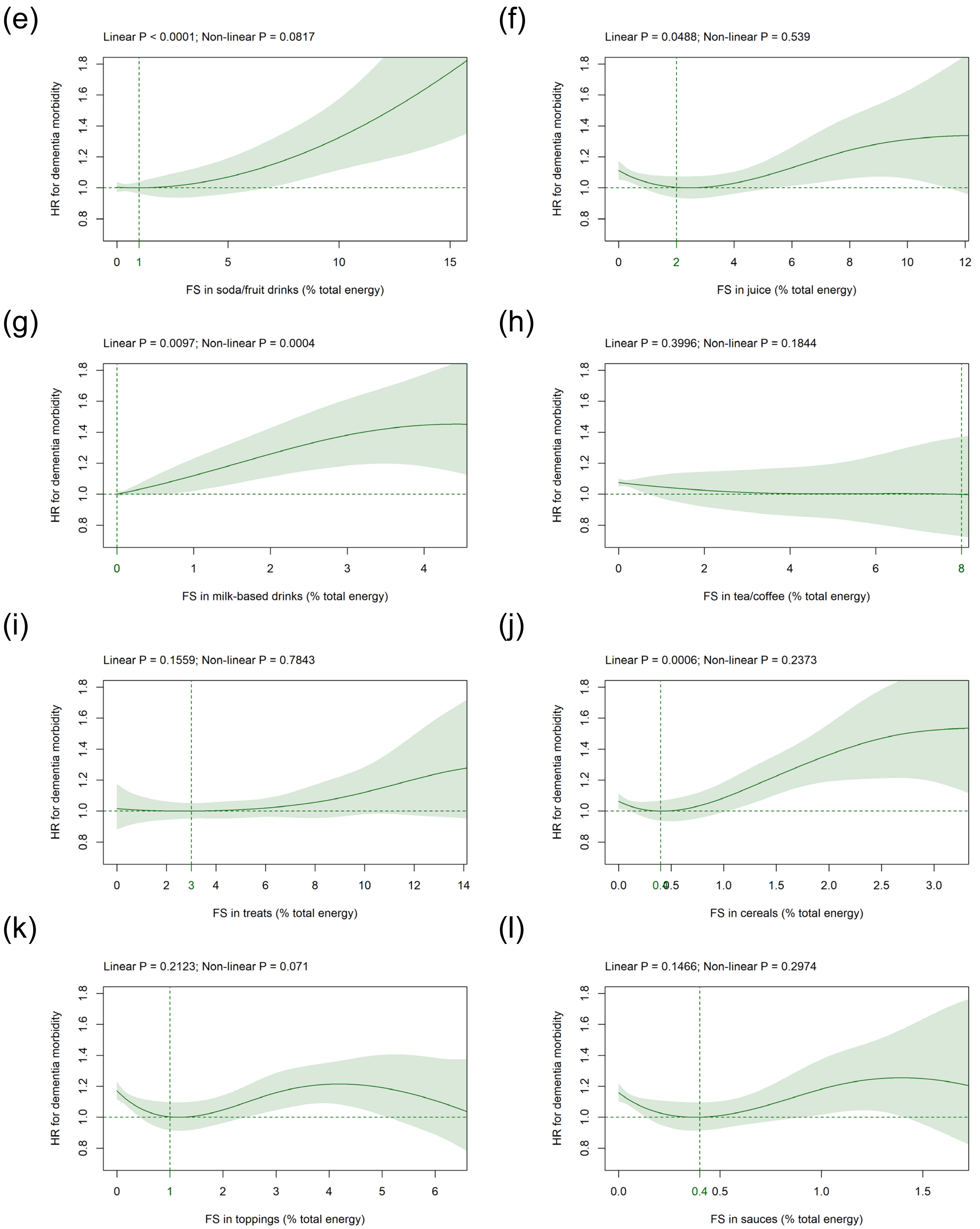

Figure S14 continued

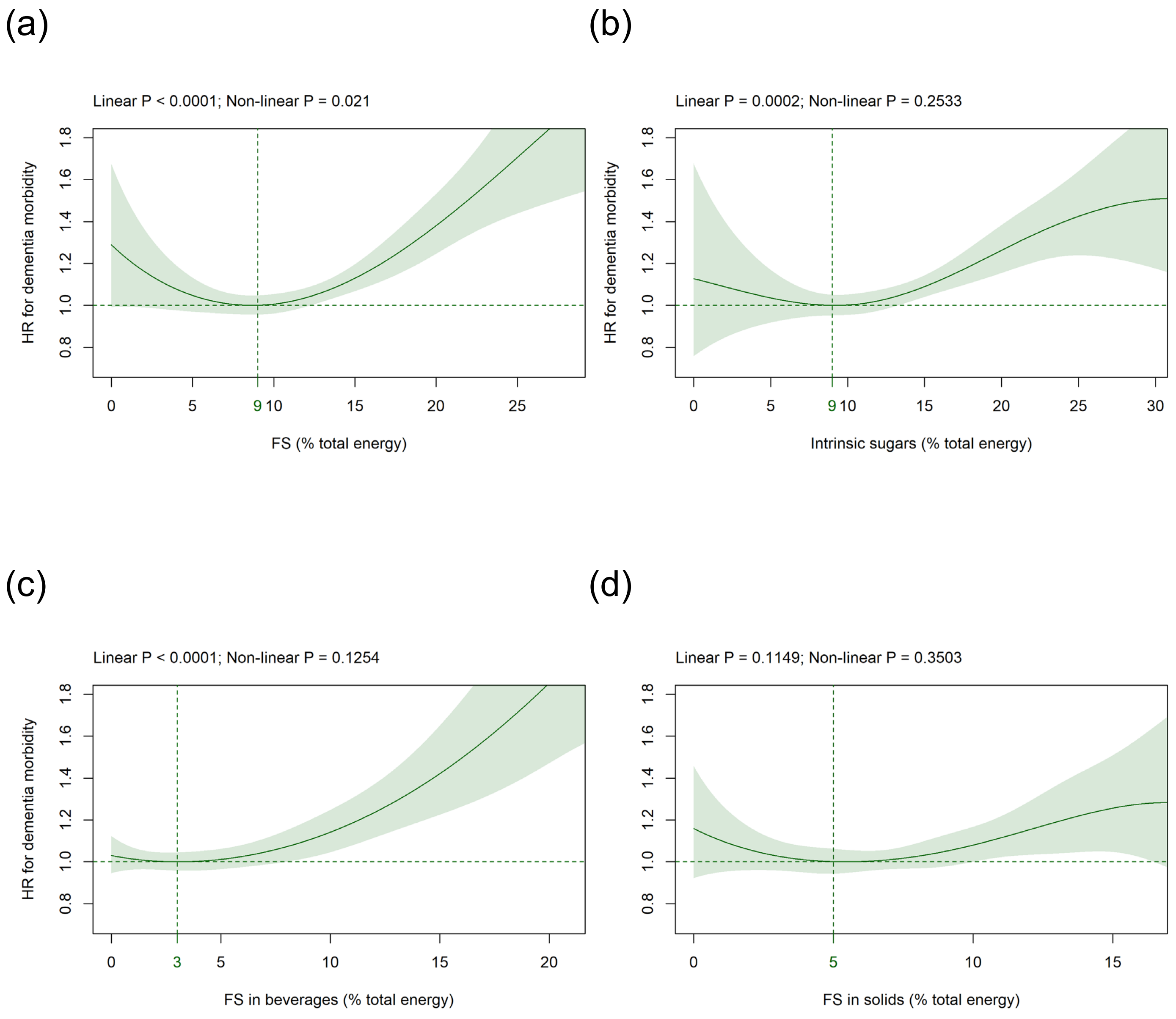
(a)
Linear P < 0.0001; Non-linear P = 0.021
HR for dementia morbidity
0.8
1.0
1.2
1.4
1.6
1.8
0
5
9
10
15
20
25
FS (% total energy)
(b)
Linear P = 0.0002; Non-linear P = 0.2533
HR for dementia morbidity
0.8
1.0
1.2
1.4
1.6
1.8
0
5
9
10
15
20
25
30
Intrinsic sugars (% total energy)
(c)
Linear P < 0.0001; Non-linear P = 0.1254
HR for dementia morbidity
0.8
1.0
1.2
1.4
1.6
1.8
0
3
5
10
15
20
FS in beverages (% total energy)
(d)
Linear P = 0.1149; Non-linear P = 0.3503
0.8
1.0
1.2
1.4
1.6
1.8
0
5
10
15
FS in solids (% total energy)

Figure S15

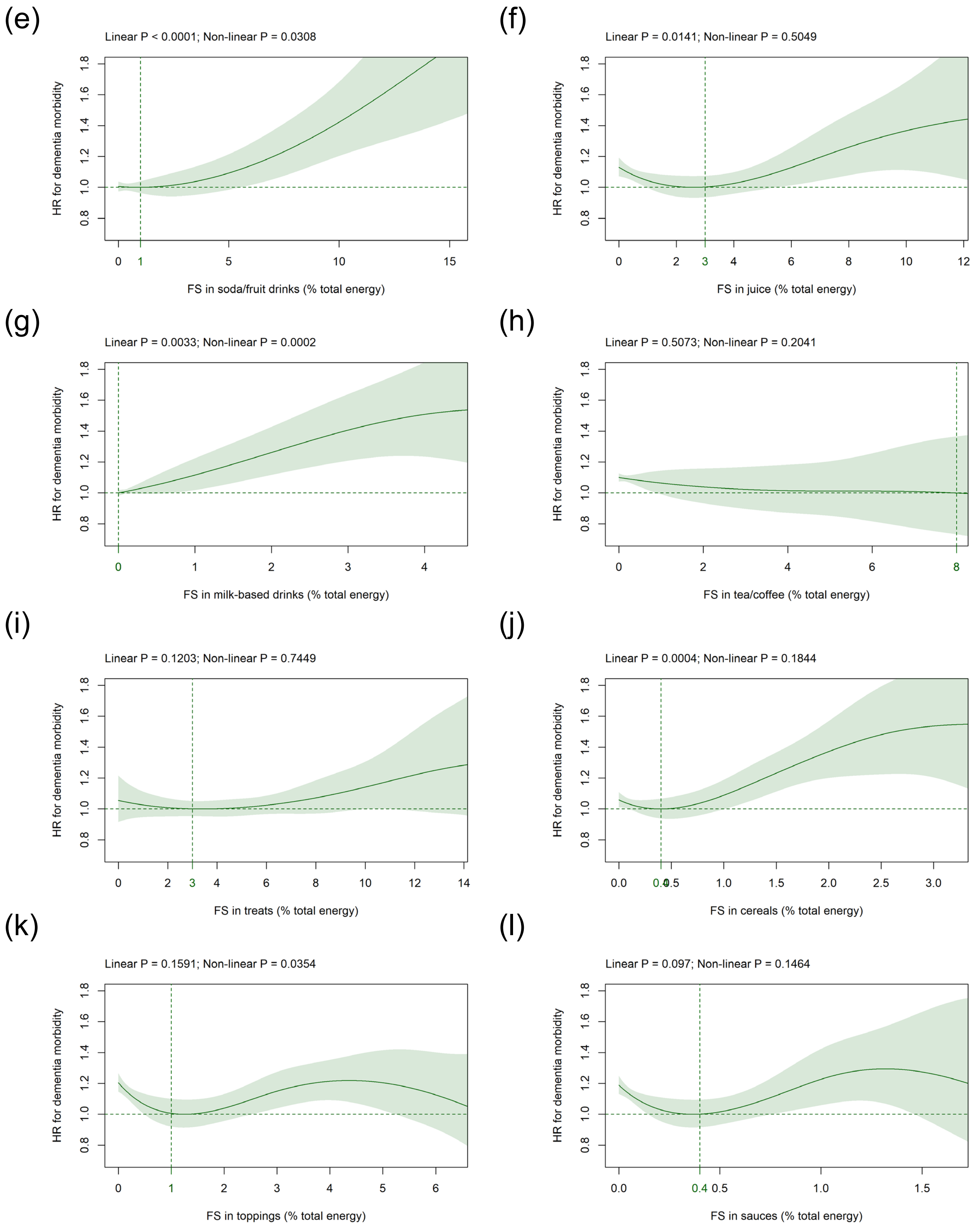

Figure S15 continued

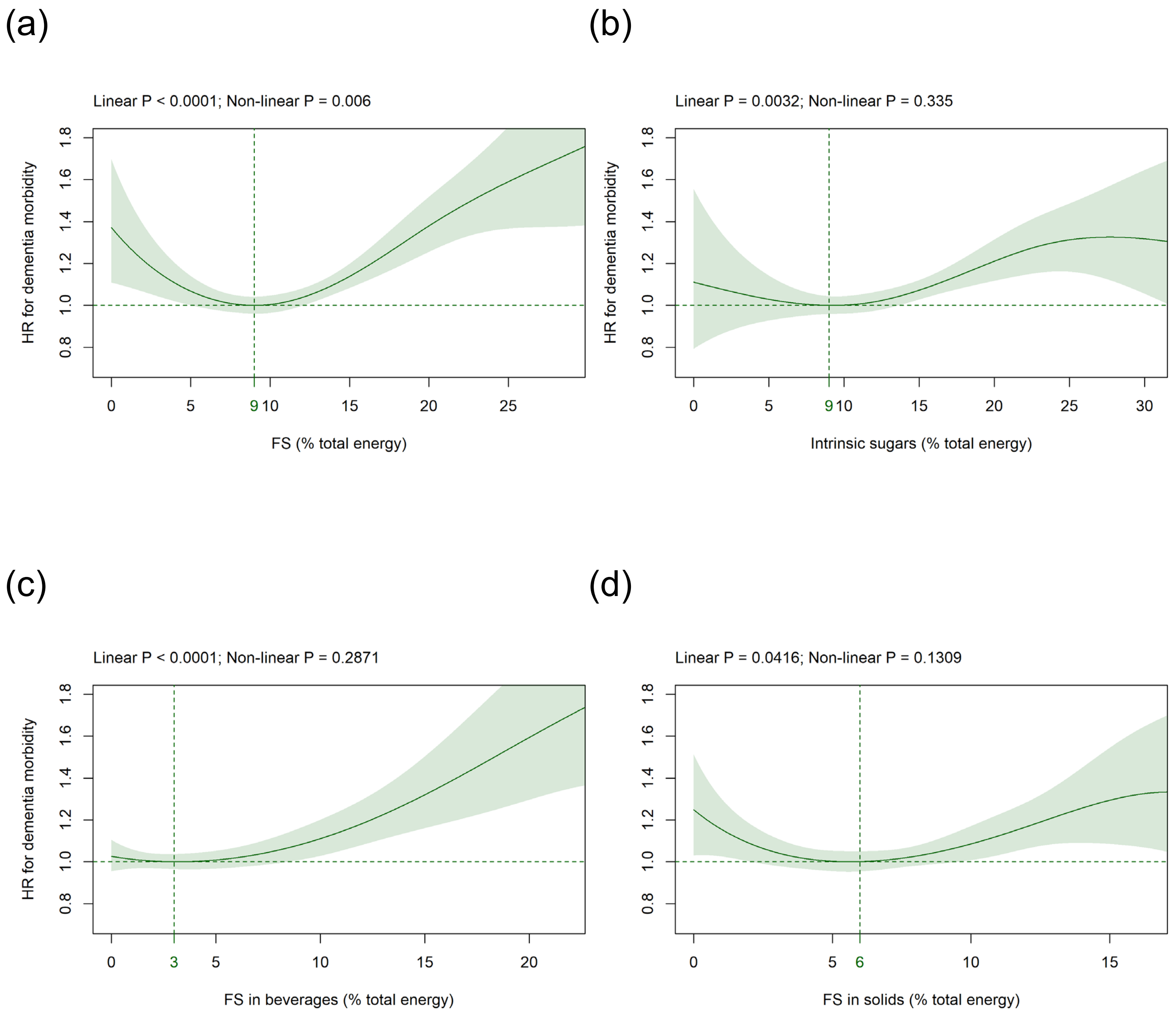

(a)
Linear P < 0.0001; Non-linear P = 0.006
HR for dementia morbidity
0.8
1.0
1.2
1.4
1.6
1.8
0
5
9
10
15
20
25
FS (% total energy)
(b)
Linear P = 0.0032; Non-linear P = 0.335
HR for dementia morbidity
0.8
1.0
1.2
1.4
1.6
1.8
0
5
9
10
15
20
25
30
Intrinsic sugars (% total energy)
(c)
Linear P < 0.0001; Non-linear P = 0.2871
HR for dementia morbidity
0.8
1.0
1.2
1.4
1.6
1.8
0
3
5
10
15
20
FS in beverages (% total energy)
(d)
Linear P = 0.0416; Non-linear P = 0.1309
0.8
1.0
1.2
1.4
1.6
1.8
0
5
6
10
15
FS in solids (% total energy)

Figure S16

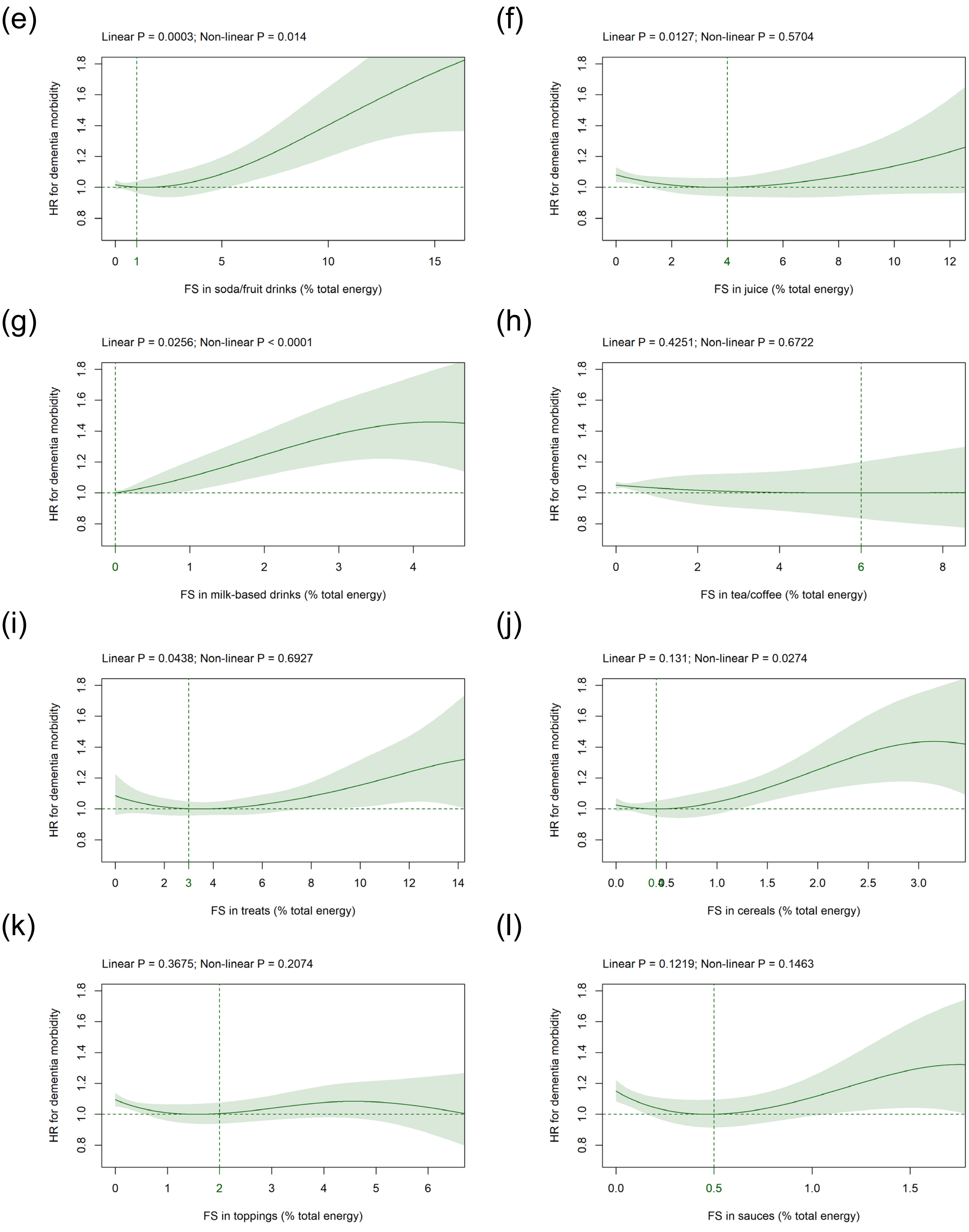

Figure S16 continued
